# Platelet miRNA-Based Signature as a Potential Non-Invasive Tool for Discriminating Patients with Secondary Progressive Multiple Sclerosis from Healthy Individuals

**DOI:** 10.3390/ijms27219914

**Published:** 2026-11-05

**Authors:** Angela Dziedzic, Michał Seweryn Karbownik, Michał Arabski, Elżbieta Miller, Sylwia Michlewska, Joanna Saluk

**Affiliations:** 1 Department of General Biochemistry, Faculty of Biology and Environmental Protection, University of Lodz, Pomorska 141/143, 90-236 Lodz, Poland; joanna.saluk@biol.uni.lodz.pl; 2 Department of Pharmacology and Toxicology, Medical University of Lodz, Zeligowskiego 7/9, 90-752 Lodz, Poland; michal.karbownik@umed.lodz.pl; 3 Division of Medical Biology, Institute of Biology, Jan Kochanowski University, Uniwersytecka 7, 25-406 Kielce, Poland; arabski@ujk.edu.pl; 4 Department of Neurological Rehabilitation, Medical University of Lodz, Milionowa 14, 93-113 Lodz, Poland; elzbieta.dorota.miller@umed.lodz.pl; 5 Laboratory of Microscopic Imaging and Specialized Biological Techniques, Faculty of Biology and Environmental Protection, University of Lodz, Banacha 12/16, 90-237 Lodz, Poland; sylwia.michlewska@biol.uni.lodz.pl

**Keywords:** miRNA, let-7a-5p, miR-146a-5p, biomarker, non-invasive tool, blood platelets, megakaryocytes, multiple sclerosis

## Abstract

Multiple sclerosis (MS) lacks minimally invasive biomarkers that reliably reflect disease-associated pathophysiological alterations, particularly during the progressive stage of the disease. Because blood platelets contribute to thrombo-inflammatory signalling and are the major vascular carriers of regulatory miRNA, we investigated whether platelet miRNA alterations are associated with platelet hyperreactivity in MS and whether these changes originate during thrombopoiesis by examining megakaryocytes. We analyzed platelets and megakaryocytes from MS patients and healthy controls by transmission electron microscopy, flow cytometry, ELISA, miRNA profiling based on microarrays, and Real-Time PCR validation. Logistic regression and bioinformatic analyses were used to evaluate the potential and biological relevance. MS platelets exhibited a hyperreactive phenotype, characterized by enhanced degranulation, microparticle release, P-selectin expression, and collagen-induced aggregation. GPVI levels were significantly elevated in platelets but remained unchanged in megakaryocytes. MiRNA profiling revealed widespread downregulation in platelets, while megakaryocytes showed selective upregulation of miR-223-3p. Following microarray screening, PCR validation of 10 selected miRNAs revealed widespread downregulation in platelets, while only miR-223-3p was significantly upregulated in megakaryocytes from MS patients. A two-miRNA model (let-7a-5p and miR-146a-5p) showed good internal discrimination (AUC = 0.89; sensitivity 89%; specificity 85%), but lower external performance (C-index = 0.66; sensitivity 50%; specificity 73%). MS is associated with a distinct platelet miRNA signature linked to acquired platelet hyperreactivity and vesiculation. Platelet-derived let-7a-5p and miR-146a-5p represent potential non-invasive biomarker candidates, although their diagnostic utility requires further validation in larger independent cohorts.

## 1. Introduction

Multiple sclerosis (MS) is a chronic autoimmune and neuroinflammatory disease of the central nervous system (CNS), characterized by disruption of the blood–brain barrier (BBB), infiltration of immune cells, demyelination, and progressive neurodegeneration. The clinical course of MS is heterogeneous, encompassing relapsing–remitting (RRMS) and progressive forms, including primary progressive (PPMS) and secondary progressive MS (SPMS), which differ in their patterns of disease activity, disability accumulation, and progression [1]. Although magnetic resonance imaging (MRI) and the detection of oligoclonal bands in cerebrospinal fluid are central to the diagnosis of MS, these approaches provide limited insight into the molecular mechanisms underlying disease progression [2]. Therefore, identifying minimally invasive molecular biomarkers that reflect systemic pathophysiological alterations associated with MS remains an unmet clinical need.

Growing evidence suggests that MS is not only a CNS-restricted disorder, but also involves systemic vascular and thrombo-inflammatory dysregulation, including altered interactions between circulating blood components and the neurovascular unit [3]. Platelets are increasingly recognized as active regulators of inflammation and vascular integrity, with functions that extend well beyond their traditional role in hemostasis. Through interactions with endothelial and immune cells, platelets can modulate leukocyte recruitment and contribute to BBB dysfunction, processes central to the pathophysiology of MS [4]. Despite increasing recognition of the role of platelets in neuroinflammation, studies specifically addressing platelet biology in MS remain extremely limited, highlighting this area as an emerging and underexplored research niche. Evidence of enhanced platelet activation in MS has been reported only in a few studies [5,6], including our investigations [7,8,9,10,11,12].

Platelet-derived microRNAs (miRNAs) have emerged as promising but largely unexplored biomarker candidates. Despite being anucleate, platelets retain a complex molecular machinery, including a rich repertoire of miRNAs that regulate gene expression post-transcriptionally. These small non-coding RNAs participate in vascular and immune signalling, while their expression profiles may reflect changes in platelet functional status [13]. Platelet-derived miRNAs can also be released within extracellular vesicles, facilitating intercellular communication and potentially modulating the endothelial and immune cell response. Thus, platelet miRNAs may serve not only as molecular indicators of disease-associated alterations but also as functional mediators of thrombo-inflammatory processes [14,15,16].

In the present study, our aim was to identify a platelet miRNA signature with potential utility as a non-invasive tool to discriminate patients with SPMS from healthy individuals. Therefore, we profiled miRNA expression in platelets and their precursor cells (megakaryocytes) of patients with MS and healthy controls using a two-stage approach combining microarray screening with Real-Time PCR validation. In parallel, we characterized platelet ultrastructure and activation status using transmission electron microscopy (TEM) and flow cytometry to investigate whether alterations in the miRNA profile are associated with platelet dysfunction. By integrating molecular, functional, and bioinformatic analyses, we identified an MS-associated platelet miRNA signature accompanied by a hyperreactive platelet phenotype. We further evaluated the discriminatory performance of selected miRNAs, supporting their potential role as a non-invasive biomarker for distinguishing SPMS patients from healthy individuals.

## 2. Results

### 2.1. Signatures of Intensified Platelet Vesiculation and Cargo Secretion in MS

The ultrastructural platelet morphology was qualitatively assessed in samples obtained from three MS patients and three control subjects. Representative micrographs were acquired from independent, non-overlapping fields of view at different magnifications to ensure a broader morphological assessment of the platelet populations. Samples from MS patients and healthy controls were analyzed, with 2 grids per sample and at least 10 fields of view and 10–30 platelets examined per donor. Images illustrating recurrent ultrastructural features observed in each study group were selected for presentation. All TEM image acquisition and morphological assessments were performed by the same investigator.

**Figure 1:**
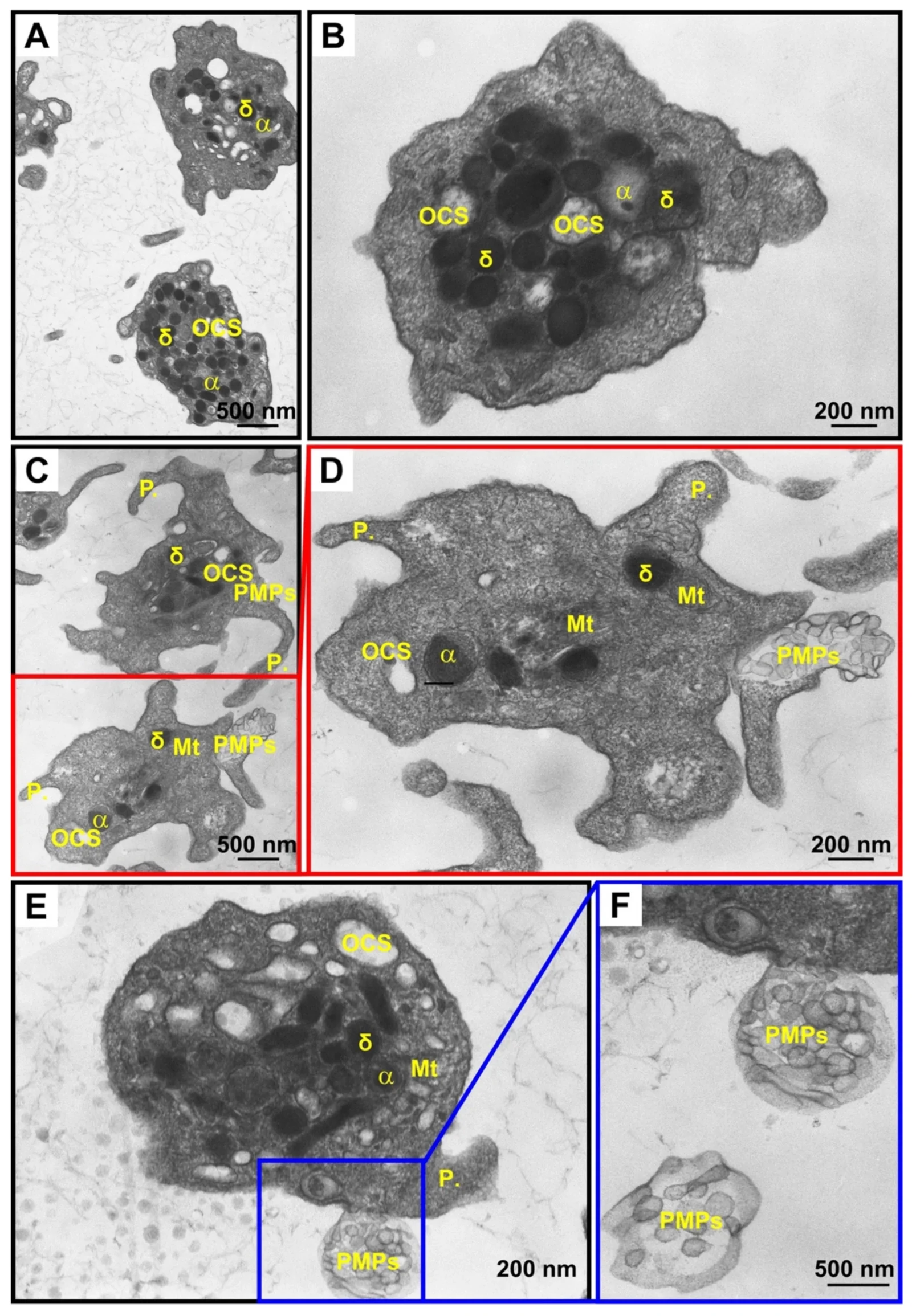
Ultrastructural changes in blood platelets from the control group (*n* = 3) (**A**,**B**) versus MS patients (*n* = 3) (**C**–**F**). Designated structures: α-granules (α), dense granules (δ), open canalicular system (OCS), mitochondria (Mt), pseudopodia (P.), and platelet-derived microparticles (PMPs). Cell ultrastructure was examined on a TEM JEM 1010 (JEOL Ltd., Tokyo, Japan) at 80 kV.

The blood platelets in the control group exhibited their characteristic discoid morphology with a well-preserved ultrastructure, consistent with a quiescent or minimally activated state. These cells displayed rounded to slightly oval profiles with intact plasma membranes (Figure 1A,B). The cytoplasm was densely packed with α-granules (α) and dense granules (δ) predominantly distributed within the central cytoplasmic region (Figure 1A,B). The open canalicular system (OCS) developed moderately, with relatively few membrane invaginations (Figure 1A,B), reflecting minimal secretory activity and retention of granule content.

Platelets from MS patients exhibited pronounced ultrastructural alterations consistent with increased platelet activation and degranulation. The most noticeable change was a marked reduction in the abundance of α-granules (α) and dense granules (δ) compared to controls (Figure 1A–E). Lower-magnification TEM images that encompassed broader fields of view further revealed a reduced intracellular granule abundance in platelets from MS patients compared to healthy controls (Supplementary Figure S1). This degranulation created numerous organelle-free zones (electron-lucent regions) throughout the cytoplasm, consistent with release of the granule contents (Figure 1C–E). Cell morphology was highly irregular with multiple pseudopodia (P) (Figure 1C–E). The OCS appeared more prominent and structurally complex, suggesting increased membrane remodeling and elevated granule secretion (Figure 1C–E). Mitochondria (Mt) were occasionally observed near the cell periphery (Figure 1D,E). Numerous platelet-derived microparticles (PMPs) were observed in the extracellular space surrounding MS platelets (Figure 1C–F). These membrane-bound structures varied in size and often contained electron-dense material or showed multivesicular architecture (Figure 1C–F), suggesting enhanced membrane vesiculation. PMPs were seen budding from pseudopodial tips and freely distributed in the medium, demonstrating ongoing shedding from the platelet surface (Figure 1C–F). Extensive PMP formation, marked granule depletion, organelle-free zones, elaborate OCS, and numerous pseudopodia can demonstrate spontaneous platelet hyperactivation and excessive degranulation in MS patients, contrasting strongly with granule-rich and minimally activated controls.

### 2.2. MS Platelets Exhibit Heightened Sensitivity to Collagen with Increased Aggregation, Vesiculation, and P-Selectin Expression

**Figure 2:**
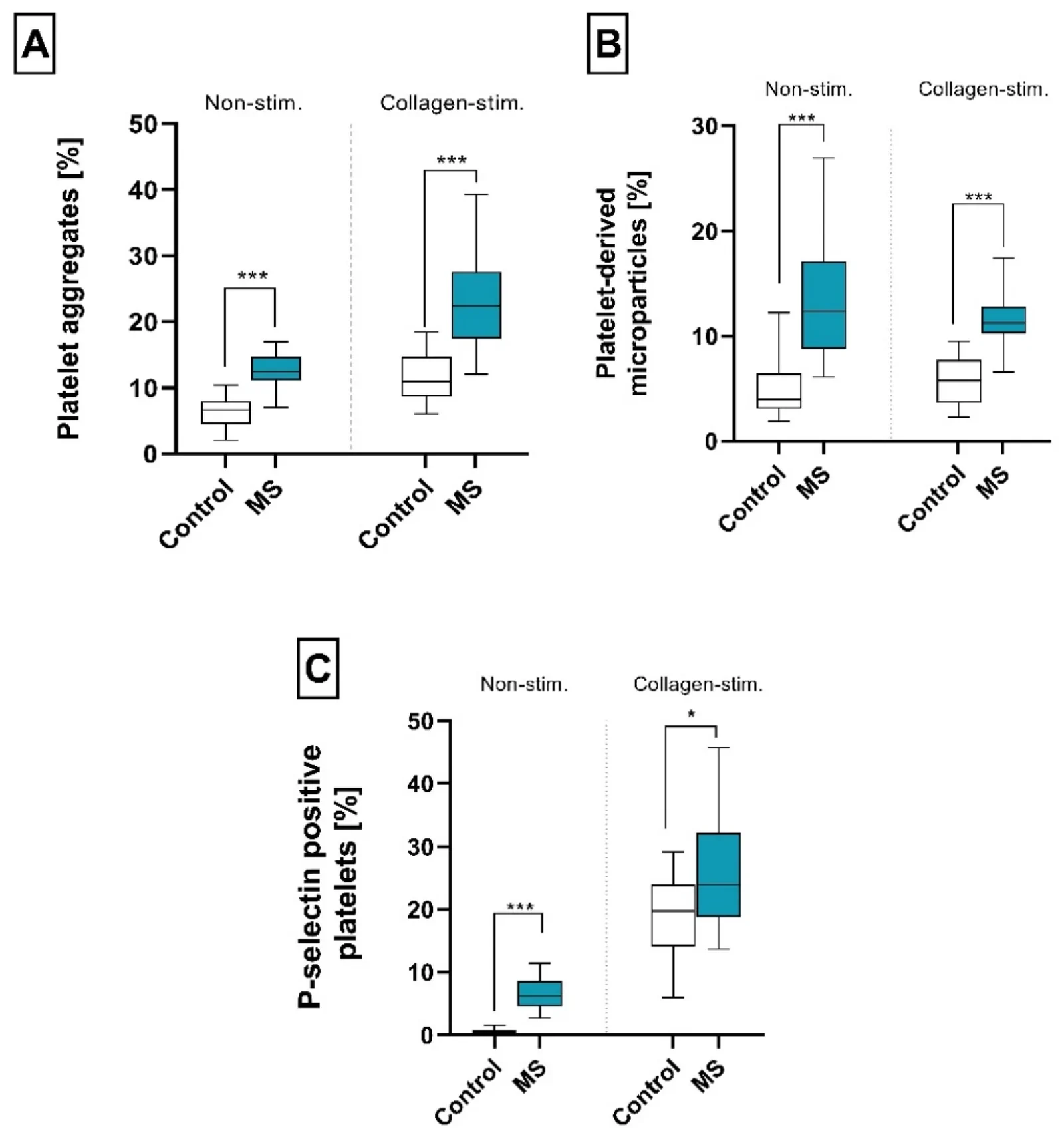
The percentage of platelet aggregates (PAGs) (**A**), platelet-derived microparticles (PMPs) (**B**), and expression of P-selectin on platelets (**C**). Results are expressed as the percentage of the total platelet population, defined as 15,000 CD61-positive events. Measurements were performed under unstimulated conditions and after collagen stimulation (1 μg/mL), using standardized gating and acquisition settings. CD61-positive objects with log-scale FSC above 10^3.5^ were classified as PAGs, whereas events with FSC below 10^2.2^ were classified as PMPs. P-selectin expression on CD61/FITC-positive cells was assessed using CD62/PE fluorescence, and the percentage of CD61+CD62P+ platelets was calculated relative to the total platelet count (15,000 CD61+ events). Data were acquired using the Cube 6 cytometer and analyzed with CyView Software v.1.5.5.8 (Sysmex Partec, Görlitz, Germany). The central line represents the median, boxes indicate the Q1 and Q3, and whiskers denote the min. and max. values. Statistical analysis was performed using the Mann–Whitney U test; * *p* < 0.05, *** *p* < 0.001.

Flow cytometric analysis revealed a markedly enhanced activation state and reactivity of circulating blood platelets in MS patients compared to controls in both resting conditions (non-stimulated) and collagen-stimulated conditions (Figure 2). Collagen was selected as a physiologically relevant platelet agonist that induces activation primarily through GPVI-mediated signalling. A relatively low concentration (1 µg/mL) was applied to assess platelet responsiveness while limiting the probability of maximal activation. This approach was particularly relevant given the involvement of GPVI signalling in platelet degranulation and extracellular vesicle release, including the release of platelet-associated miRNAs [17,18,19].

In the absence of exogenous stimulation, MS platelets exhibited a markedly activated phenotype, characterized by an approximately 2-fold increase in platelet aggregates (PAGs) (12.8% vs. 6.6%; *p* < 0.0001) (Figure 2A), a more than 3-fold increase in the number of platelet-derived microparticles (PMPs) (12.4% vs. 4.0%; *p* < 0.0001) (Figure 2B), and more than 10-fold increase in the level of P-selectin–positive platelets (6.2% vs. 0.5%; *p* < 0.0001) relative to controls (Figure 2C).

Upon collagen stimulation, this hyperreactive profile persisted. The number of PAGs remained approximately twice as high in the MS group as in controls (22.4% vs. 10.9%; *p* < 0.0001) (Figure 2A), and PMP release remained almost 2-fold higher (11.3% vs. 5.8%; *p* < 0.0001) (Figure 2B). Although collagen robustly induced P-selectin expression in both groups, MS platelets still displayed significantly elevated surface levels (24.0% vs. 19.7%; *p* = 0.019) (Figure 2C).

These findings indicate a constitutive and agonist-amplified platelet hyperreactivity in MS, underscoring a potentially underappreciated contribution of platelet dysregulation to vascular and inflammatory pathology associated with MS. Flow cytometry-based evaluation of platelet activation and reactivity in MS patients and controls is summarized in Table 1, while the corresponding gating strategy is presented in Supplementary Figure S2.

### 2.3. Increased GPVI Expression Drives a Pro-Adhesive Phenotype in MS Platelets

To explore the molecular basis of this pro-adhesive phenotype, we quantified the expression of glycoprotein VI (GPVI), a major platelet collagen receptor. To investigate whether alterations in platelet GPVI levels are also present in their precursor cells, or emerge later through epigenetic or maturation-related changes in circulating platelets, we examined GPVI expression in both platelets and megakaryocytes from MS patients and the control group.

**Figure 3:**
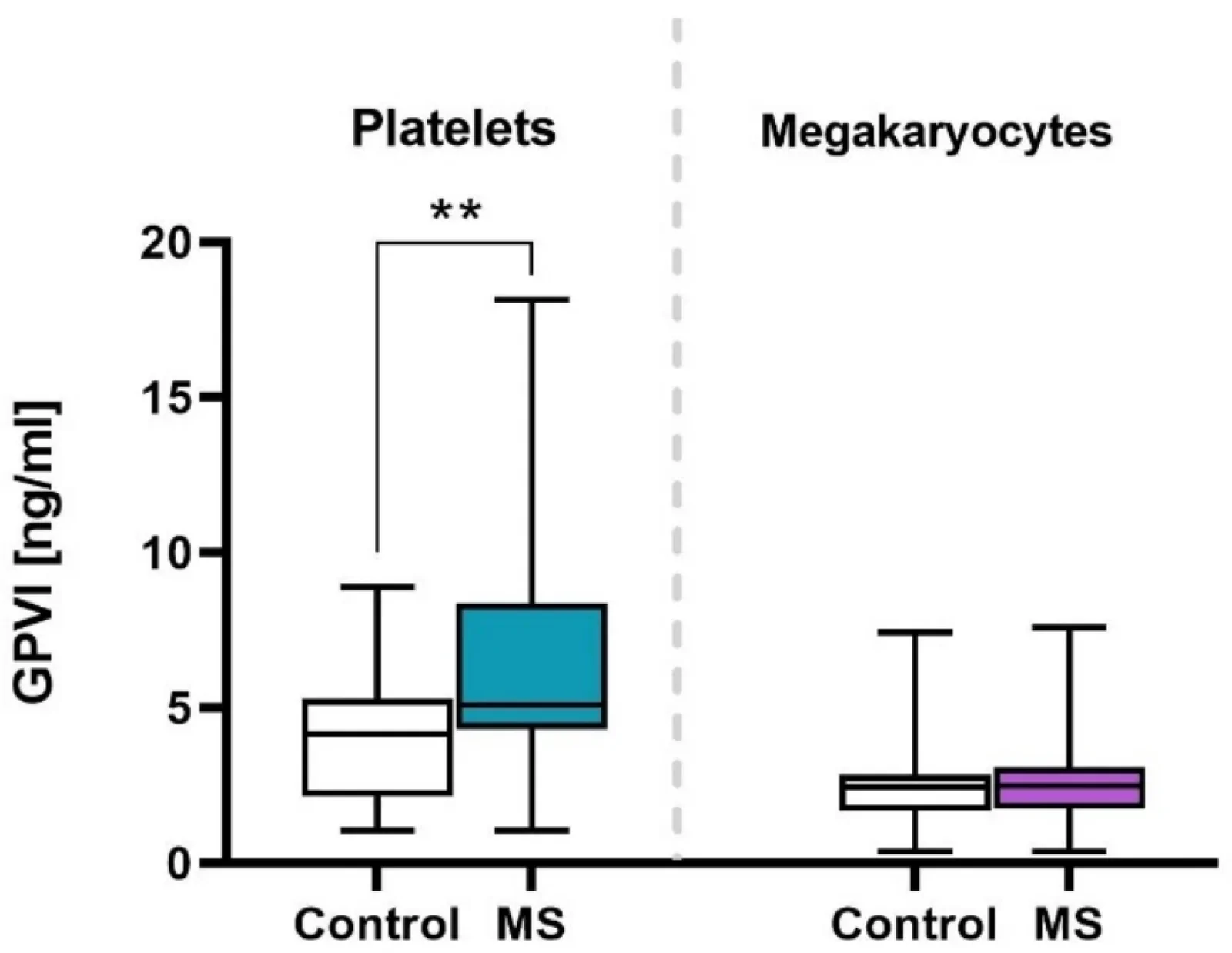
GPVI concentrations (ng/mL) in platelets and megakaryocytes from MS patients (*n* = 26) and the control group (*n* = 26). Data are presented as median with Q1 and Q3 (whiskers denote the min. and max. values). Statistical analysis was performed using the Mann–Whitney U test; ** *p* < 0.01.

ELISA analysis demonstrated a higher concentration of GPVI in platelet lysates from MS patients compared to controls, while no significant differences were observed in megakaryocytes (Figure 3). The mean platelet GPVI concentration in MS was 1.65-fold higher than in controls (6.75 ± 4.32 ng/mL vs. 4.09 ± 2.13 ng/mL, respectively; *p* = 0.0086). In contrast, GPVI concentrations in megakaryocytes did not differ significantly between MS patients and controls (2.50 ± 1.58 ng/mL vs. 2.40 ± 1.37 ng/mL, respectively) (Figure 3).

These findings indicate that elevated platelet GPVI levels in MS are not accompanied by corresponding changes in megakaryocytes, suggesting that platelet-associated alterations may arise independently of detectable differences in their precursor cells. Increased GPVI levels may contribute to the enhanced collagen responsiveness observed in platelets from MS patients.

### 2.4. Microarray-Based Screening Analysis of the Platelet and Megakaryocyte miRNome

Given the role of PMPs as carriers of miRNA, we hypothesized that altered miRNA expression may contribute to platelet hyperreactivity and serve as a potential biomarker of platelet activation. To investigate this, we performed microarray-based screening of miRNA profiles in platelets and their precursor cells, megakaryocytes, to identify differentially expressed miRNAs for subsequent RT-qPCR validation.

**Figure 4:**
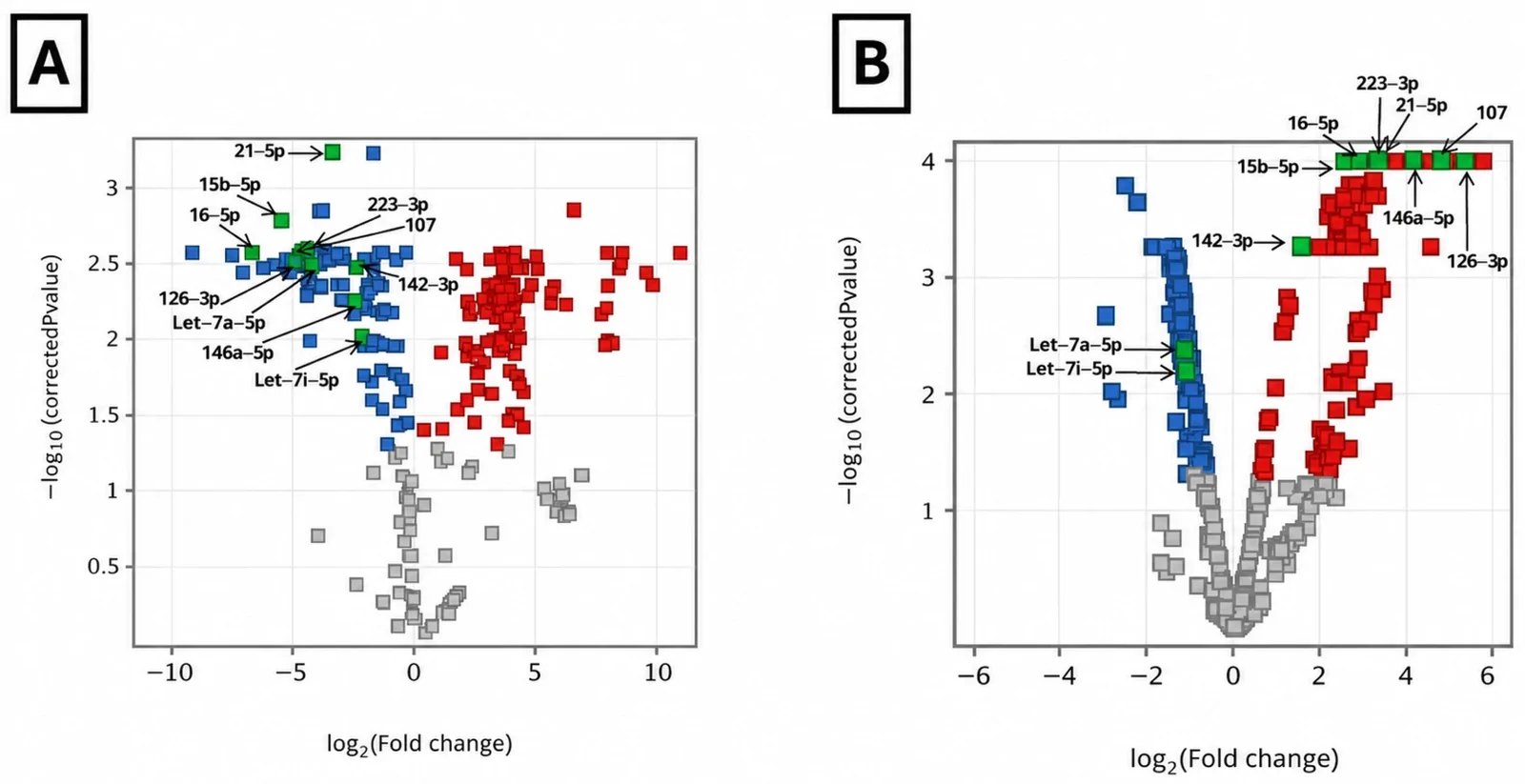
Volcano plots depicting the relationship between the negative logarithm of the FDR-adjusted *p*-value (Benjamini–Hochberg correction; Y-axis) and the FC logarithm (FC; X-axis) in miRNA expression profiles obtained from microarray analysis in blood platelets (**A**) and megakaryocytes (**B**), comparing MS patients with the control group. MiRNAs exhibiting a fold change greater than two (|FC| > 2) are highlighted in blue (downregulated) and red (upregulated), while grey points represent non-significant transcripts (FDR-adjusted *p* > 0.05). Green squares indicate selected differentially expressed miRNAs subjected to further validation by Real-Time PCR.The plots were generated using the GeneSpring software (version 14.9.1; Agilent Technologies, Santa Clara, CA, USA).

Microarray analysis revealed substantial dysregulation of miRNA expression in platelets and megakaryocytes from MS patients compared to controls. It was performed as an exploratory screening approach using a moderated *t*-test with Benjamini–Hochberg correction for multiple comparisons (FDR-adjusted *p*-value < 0.05 and FC ≥ 2). In platelets, 130 miRNAs increased significantly and 67 decreased significantly in MS compared to controls. In megakaryocytes, 124 miRNAs showed increased expression, and 77 showed decreased expression in MS compared to the control group. Differential expression across all identified miRNAs from blood platelets and megakaryocytes is presented on characteristic volcano plots, displaying –log(corrected *p*-value) against log2(fold change) (Figure 4). The complete list of identified miRNAs is provided in Supplementary Table S1.

Among the miRNAs identified by microarray screening, only 10 met all the predefined selection criteria, including significantly altered expression in platelets and megakaryocytes of MS patients compared to controls (Benjamini–Hochberg-adjusted *p* < 0.05 and FC ≥ 2) and well-documented functional relevance to platelet biology (Table 2). The primary biological criterion was the previously documented involvement of these miRNAs in processes directly related to the platelet functional parameters investigated in our study, particularly GPVI-mediated platelet activation, collagen-dependent adhesion, and platelet-derived microparticle release. These mechanisms may contribute to the improved prothrombotic platelet phenotype observed in progressive MS. Thus, the combined application of statistical and biological criteria resulted in the identification of 10 miRNAs that met all selection requirements and were subsequently subjected to Real-Time PCR validation.

### 2.5. Real-Time PCR miRNA Analysis

The expression profiles of the 10 miRNAs selected from the initial microarray screening were subsequently validated using Real-Time PCR in blood platelets and megakaryocytes from MS patients and healthy controls.

**Figure 5:**
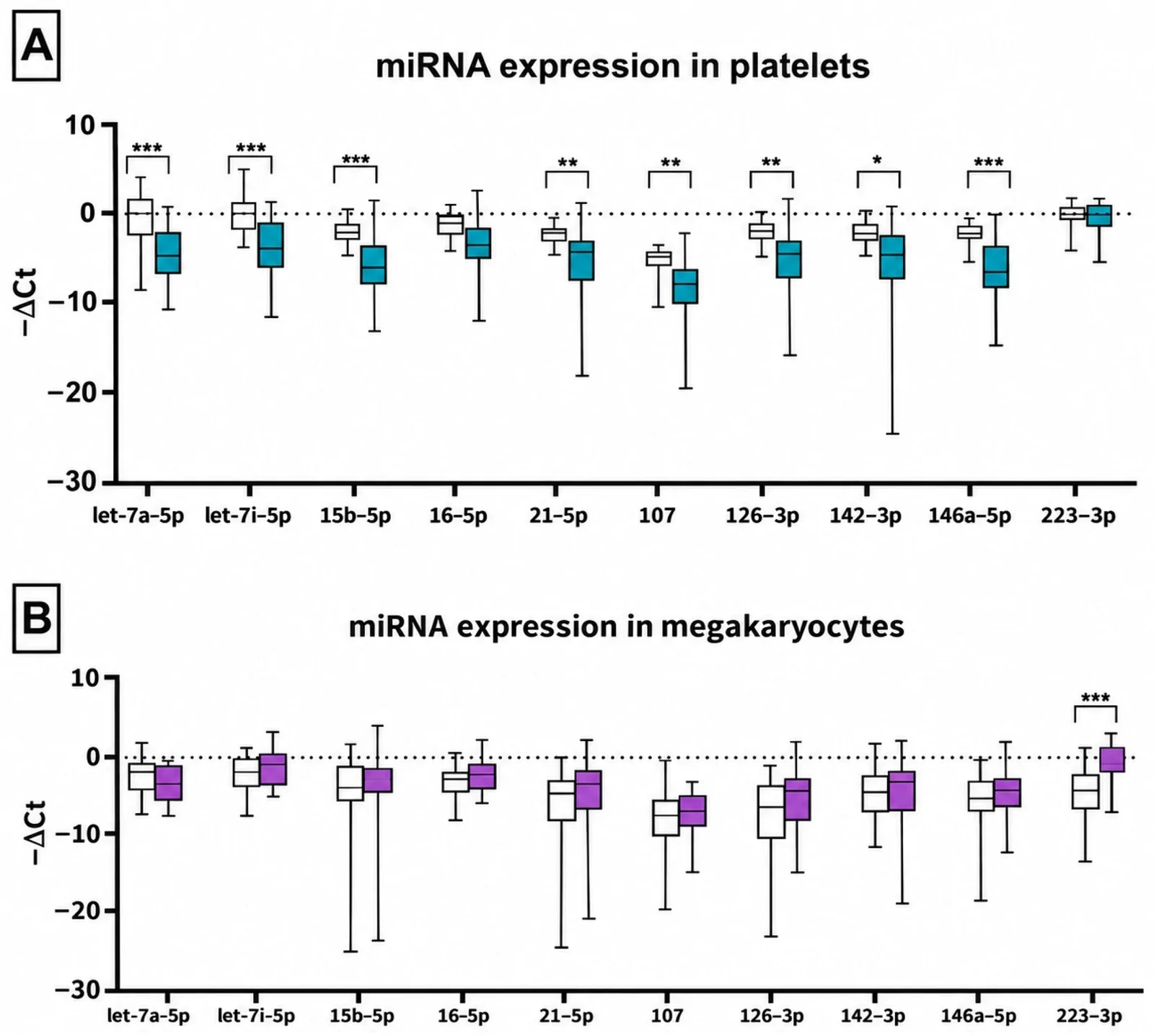
Expression of miRNAs (let-7a-5p, let-7i-5p, 15b-5p, 16-5p, 107, 126-3p, 142-3p, 146a-5p, 223-3p) measured by Real-Time PCR in platelets (**A**) and megakaryocytes (**B**) in the MS group (*n* = 26) and the control group (*n* = 26)—colourless bars. The results are expressed as median −ΔCt (with Q1 and Q3; whiskers min. and max. values) relative to the median value of the exogenous control (*cel*-miR-39-3p) and the endogenous control (miR-191-5p). Statistical analysis was performed using the Mann–Whitney U test (corrected *p*-value), * *p* < 0.05; ** *p* < 0.01; *** *p* < 0.0001.

In blood platelets, all examined miRNAs except 223-3p showed substantially lower −ΔCt values in the MS group compared to controls, corresponding to markedly reduced expression, with the greatest decrease observed for let-7a-5p (approximately 19-fold). Adjustment for sex, age, BMI, and platelet count yielded effect sizes comparable to those obtained in unadjusted analyses, with statistical significance maintained for all miRNAs except miR-16-5p (Figure 5A).

Unlike platelets, most of the miRNAs examined showed no statistically significant differences in megakaryocyte expression between MS patients and healthy controls. The only exception was miR-223-3p, which exhibited approximately 10-fold higher expression in the MS group in both unadjusted and covariate-adjusted analyses. Additionally, miR-126-3p showed a borderline increase in expression (Figure 5B).

Table 3 summarises the miRNA expression profiles in platelets and megakaryocytes from MS patients and controls. Data are presented as mean −ΔCt values and corresponding FC.

### 2.6. Intercorrelation and Exploratory Factor Analysis (EFA)

**Figure 6:**
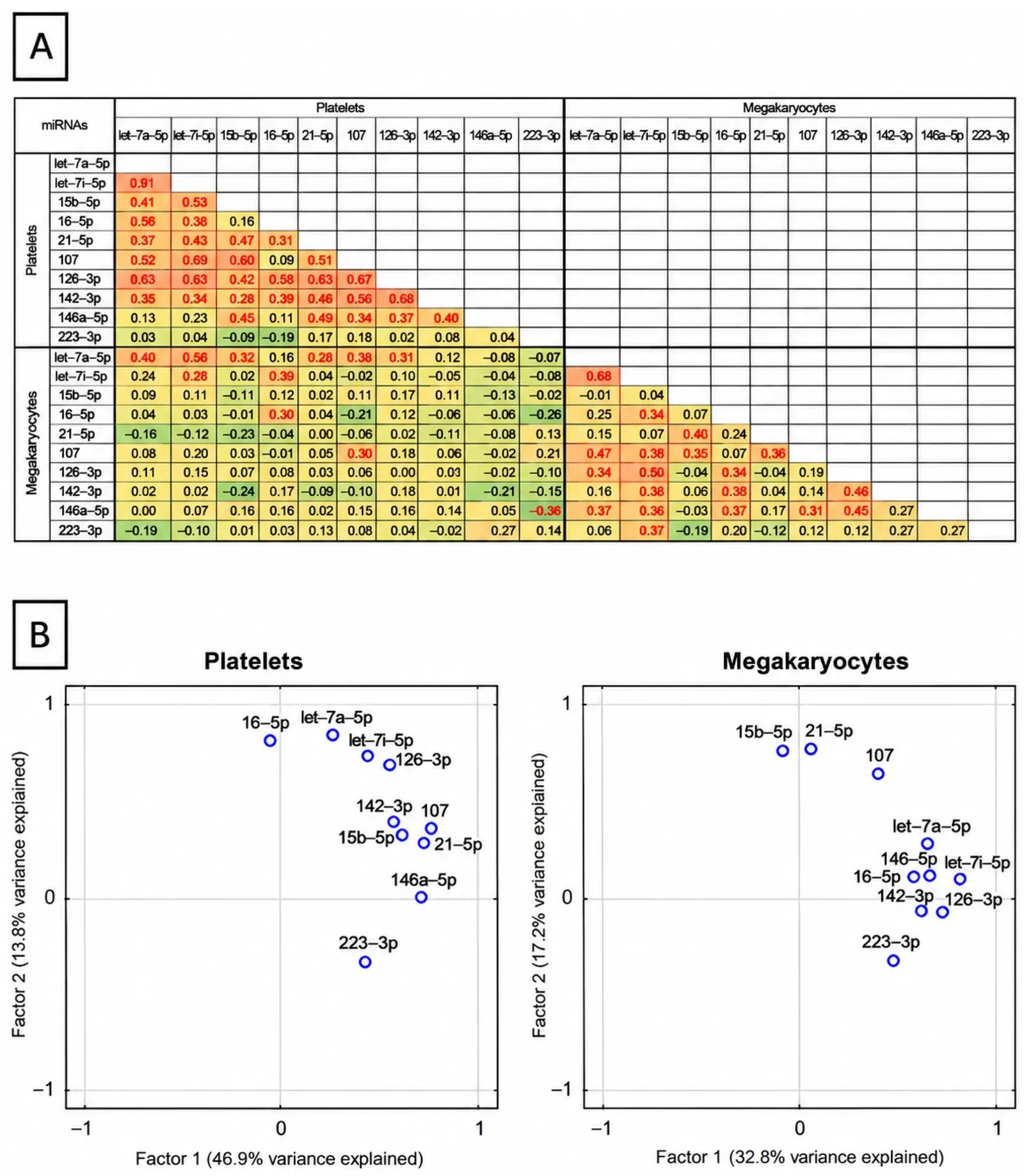
Inter-miRNA correlation structure (**A**) and exploratory factor analysis (EFA) of expression profiles (**B**) in platelets and megakaryocytes. The color gradient in the correlation matrix represents the strength and direction of correlations, ranging from green (negative correlations) through yellow (weak or near-zero correlations) to orange (strong positive correlations). Red values indicate statistically significant correlations.

The miRNAs examined exhibited significant intercorrelations in both cell types, as illustrated by the correlation matrices (Figure 6A). Kaiser–Meyer–Olkin (KMO) measure indicated acceptable sampling for blood platelet data (KMO = 0.72; Bartlett’s χ^2^(45) = 328.5, *p* < 0.0001) and lower adequacy for megakaryocyte data (KMO = 0.64; χ^2^ (45) = 144.1, *p* < 0.0001).

Two factors accounted for 60.6% and 50.0% of the total variance in platelets and megakaryocytes, respectively. The factor-loading plots (Figure 6B) revealed a more concentrated distribution of miRNA loading along Factor 1 in megakaryocytes, whereas platelet miRNAs were more widely dispersed across both factors. Although the platelet dataset showed greater suitability for factor analysis, as indicated by its higher KMO value, megakaryocyte miRNAs exhibited a more uniform distribution of factor loadings. In platelets, miR-16-5p, let-7a-5p, and let-7i-5p showed relatively stronger loadings on Factor 2, while the remaining miRNAs loaded predominantly on Factor 1; in megakaryocytes, most miRNAs showed relatively greater loadings on Factor 1, with only miR-15b-5p and miR-21-5p shifting toward Factor 2. MiR-223-3p exhibited a distinct loading pattern in both cell types.

### 2.7. Logistic Regression Analysis

The expression of let-7a-5p and miR-146a-5p in platelets was examined as potential predictive values to differentiate MS patients from controls. Not only were the two miRNAs significantly downregulated in MS patients, but they were also insignificantly intercorrelated in patients and controls: r = 0.11, *p* = 0.60 and r = 0.28, *p* = 0.17, respectively; this means both could be efficiently included in the model (Table 4).

**Figure 7:**
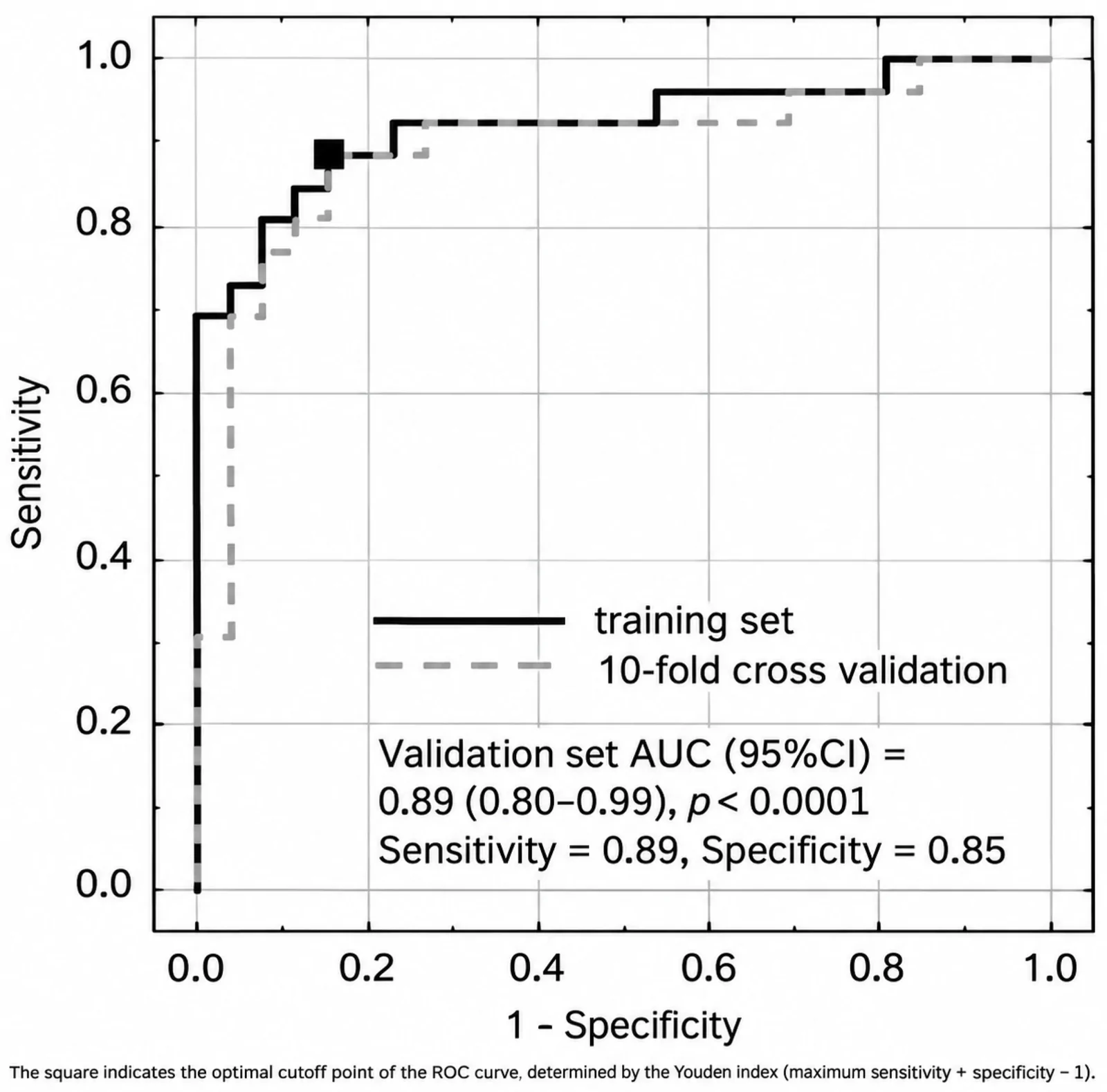
ROC curve for logistic model based on platelet-derived let-7a-5p together with miR-146a-5p differentiating MS patients from healthy controls. The internal validation of the model was performed with a 10-fold cross-validation technique. The cut-off point was proposed to be 0.482 based on the maximization of Youden’s index. The sensitivity and specificity of the internally validated model, as well as AUC with 95% CI, and the tests for AUC differences from 0.5 are displayed.

A bivariate logistic regression model with platelet let-7a-5p and miR-146-5p could explain at least half of the variance in the classification of subjects between patients with MS and controls (Cox-Snell R2 = 0.50, Nagelkerke R2 = 0.67). It reached satisfactory sensitivity and specificity even in an internal validation dataset of 89% and 85%, respectively; both miRNAs contributed significantly to the prediction (Figure 7). In an external validation sample of 22 MS patients and 22 controls with variable platelet count (mean ± SD 399.9 ± 155.9, min. 121, max. 623 [×10^3^/μL]), the model presented only moderate performance (Harrell’s C-index = 0.66), as it could not distinguish MS patients (sensitivity = 0.50) but was somewhat efficient in recognizing healthy people (specificity = 0.73). These findings indicate limited discriminatory performance in the external cohort, particularly due to low sensitivity, and highlight the need for further model optimization and validation.

### 2.8. KEGG Analysis with Gene Ontology (GO)

**Figure 8:**
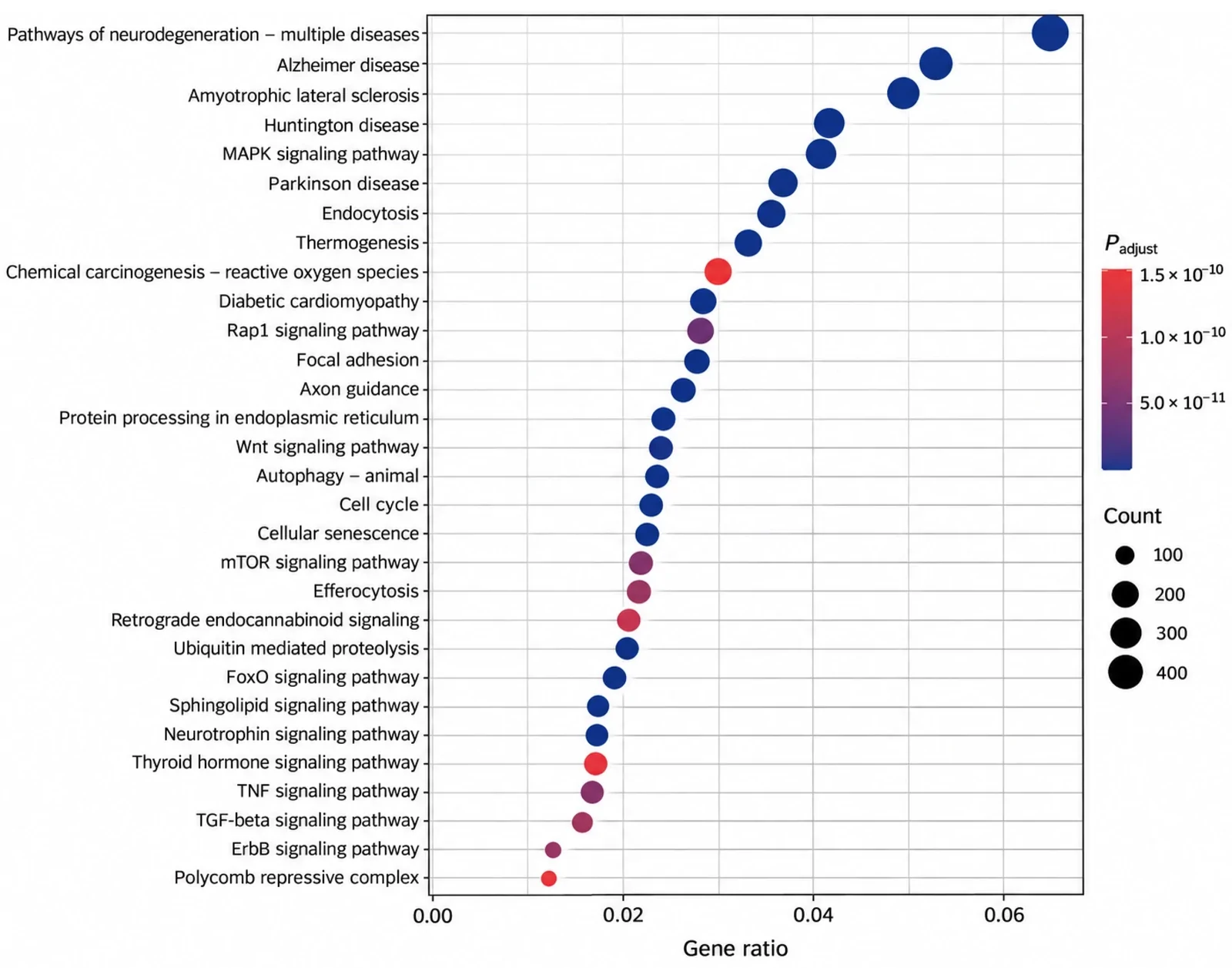
Kyoto Encyclopedia of Genes and Genomes (KEGG) pathway enrichment analysis of all study miRNAs displayed as a bubble plot. The Y-axis lists the biological pathways, while the X-axis shows the gene ratio, representing the proportion of genes associated with each pathway relative to the total number of genes analyzed. The size of the bubble reflects the number of genes assigned to each pathway (count), and the color of the bubble indicates the statistical significance after multiple-testing correction (*p*-adjust). The plot was generated using RStudio.

The most strongly enriched pathways are those related to neurodegeneration, including “Pathways of neurodegeneration—multiple diseases,” Alzheimer’s disease, amyotrophic lateral sclerosis, Huntington’s disease, and Parkinson’s disease. These exploratory findings suggest that the predicted target genes of the analyzed miRNAs may be associated with molecular pathways involved in neurodegenerative processes. Highly enriched pathways also include MAPK signalling, endocytosis, and thermogenesis. The middle portion of the plot shows pathways involved in cellular signalling and stress responses, such as “Chemical carcinogenesis—reactive oxygen species,” Rap1 signalling, focal adhesion, protein processing in the endoplasmic reticulum, Wnt signalling, and mTOR signalling. The enrichment of these pathways suggests potential associations with biological processes involved in cell survival, adhesion, responses to oxidative stress, and cellular homeostasis. Pathways with lower gene ratios and smaller counts, located at the bottom of the figure, include those related to immune and inflammatory responses, as well as hormonal regulation, for example, tumor necrosis factor (TNF), transforming growth factor β (TGF-β), neurotrophins, viral oncogene homolog erythroblastic leukemia (ErbB), and sphingolipid signalling pathways. In general, this exploratory pathway enrichment analysis identified potential associations between the predicted target genes of the examined miRNAs and biological processes related to neurodegeneration, oxidative stress, and intracellular signalling. Although these findings provide a preliminary framework to understand the possible biological relevance of the identified miRNA expression changes, they do not establish direct regulatory interactions or functional alterations in the corresponding pathways (Figure 8).

Furthermore, as part of this exploratory analysis, we identified a subset of genes commonly targeted by the analyzed miRNAs (validated by Real-Time PCR), defined as genes predicted to be regulated by at least five distinct miRNAs. The data set was curated using miRDB (https://mirdb.org accessed on 1 October 2026) (Table 5).

As part of the exploratory bioinformatic analysis, comprehensive lists of predicted mRNA targets for the 10 analyzed miRNAs were generated and annotated using Ensembl (https://www.ensembl.org/index.html accessed on 9 July 2026) and Gene Ontology (GO) (https://geneontology.org accessed on 15 July 2026) to identify candidates with potential biological relevance. From the resulting pool of predicted target genes, a subset was selected based on available literature and curated biological databases indicating possible associations with neuroinflammation, BBB dysfunction, and neurodegenerative processes implicated in MS. Subsequently, these candidate genes were prioritized based on their potential relevance to the selected pathophysiological processes.

**Figure 9:**
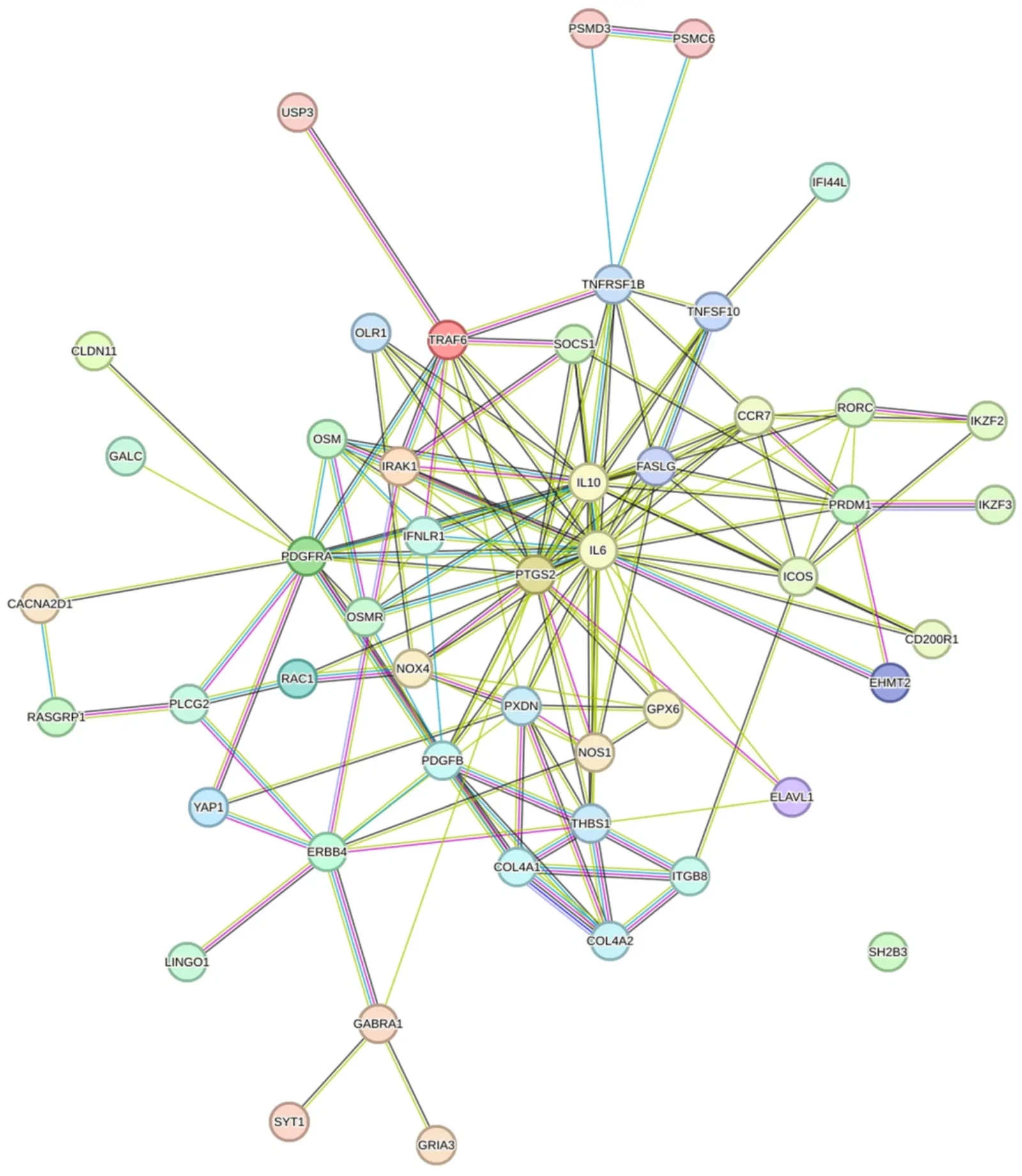
Protein–protein interaction (PPI) network of mRNA targets of two selected miRNAs, let-7a-5p and miR-146a-5p, associated with MS pathomechanisms, generated using the STRING database (https://string-db.org accessed on 30 May 2026).

To further explore potential functional associations among the predicted targets, protein–protein interaction (PPI) network analysis was performed using the STRING database (https://string-db.org accessed on 30 May 2026). This analysis focused on the predicted target genes of let-7a-5p and miR-146a-5p, the two miRNAs selected based on logistic regression and receiver operating characteristic (ROC) curve analyses (Figure 9, Table 6).

## 3. Discussion

Monitoring the progression of MS remains a major clinical challenge due to its complex pathophysiology and progressive neuroaxonal damage, often leading to irreversible disability. Current diagnostic approaches do not fully capture the dynamic nature of the disease, highlighting the need for sensitive, minimally invasive biomarkers that reflect underlying pathological processes and support patient stratification [20].

Here, we provide evidence of a distinct platelet miRNA signature potentially capable of distinguishing patients with MS patients from healthy controls, highlighting its potential as a minimally non-invasive biomarker. Importantly, this molecular signature is accompanied by functional alterations consistent with increased platelet reactivity, including collagen responsiveness (Figure 2A–C) and elevated GPVI expression (Figure 3). Complementary TEM observations revealed more pronounced platelet vesiculation and a higher occurrence of apparently empty granules in MS samples, suggesting enhanced degranulation (Figure 1C–F). Although changes in platelet shape, including pseudopodia formation, were observed in both groups and may partially reflect preanalytical activation associated with blood collection and platelet isolation, the elongated pseudopodia observed in MS samples, particularly platelets exhibiting signs of degranulation and membrane fragmentation, may indicate more advanced activation-associated alterations. These ultrastructural features were also evident in the lower-magnification overview images provided in Supplementary Figure S2. Although TEM analysis was performed on a limited number of samples (*n* = 3 per group), these preliminary observations complement the functional findings and warrant further quantitative validation in larger cohorts. Together, these findings suggest a biologically plausible association between platelet miRNA dysregulation and altered platelet function, providing a basis for further investigation of platelet-associated molecular pathomechanisms in MS.

Previous studies have demonstrated the relevance of platelet miRNAs in neurodegenerative and inflammatory disorders, providing a reason to investigate their expression in MS. Gámez-Valero et al. identified disease-associated alterations in platelet miRNA profiles in patients with Alzheimer’s disease and Lewy body dementia, including differential expression of miR-142-3p and miR-146a-5p [14]. Furthermore, miR-223 and miR-146a-5p have been linked to platelet activation and inflammatory signalling [21], while miR-223, miR-126, miR-21, and miR-146a-5p have been identified in platelet-derived extracellular vesicles [22]. These findings support the biological rationale for selecting these miRNAs for further investigation and provide a basis for interpreting the expression changes identified in our microarray analysis.

The initial microarray screening identified widespread alterations in miRNA expression profiles in platelets and megakaryocytes of MS patients. Based on predefined statistical and biological criteria, only 10 miRNAs were selected for subsequent Real-Time PCR validation, and the following discussion focuses exclusively on the expression results obtained by Real-Time PCR (Table 2).

Our Real-Time PCR validation demonstrates a significant alteration in the expression of 10 selected miRNAs in blood platelets from MS patients, with all analyzed miRNAs, except miR-223-3p, showing significantly lower levels compared to controls, reaching up to a 19-fold decrease for let-7a-5p (Figure 5A, Table 3). In contrast, among the same 10 miRNAs analyzed in megakaryocytes, only miR-223-3p showed a significant increase in expression, while the remaining miRNAs did not differ significantly between MS patients and controls (Figure 5B, Table 3). Crucially, this pattern remained largely unchanged after adjustment for possible confounders, including sex, age, BMI, and platelet count, with statistical significance retained for all miRNAs except miR-16-5p in platelets (Table 3), indicating a stable and biologically significant miRNA signature associated with MS.

The downregulation of validated miRNAs we have just observed in MS platelets suggests a change in post-transcriptional regulatory control, which may contribute to the platelet hyperreactive phenotype, which we have demonstrated in our previous studies [7,8,9,10,11,12]. Here, this interpretation is strongly supported by our ultrastructural and flow cytometric findings. This study provides the first evidence that MS platelets, distinct from the secretory phenotype characterized by enhanced degranulation (Figure 1C–F) and increased PMPs generation (Figure 2B), also have a different miRNA profile of their cargo (Figure 4A).

PMPs constitute an additional biologically active surface (approximately 100 times higher procoagulant activity than activated platelets). In particular, PMPs act as nanoscale signalling units that disseminate platelet-derived molecular cargo, including miRNAs, proteins, and lipids [23]. Due to the transfer of miRNAs to other cell types, platelet activation could regulate gene expression in endothelial and microglial sites of BBB injury. Through the expression of adhesion molecules and procoagulant factors, PMPs can recruit leukocytes, facilitate platelet–immune aggregates, and contribute to endothelial dysfunction [24]. PMPs preferentially localise to sites of vascular injury and to compromised BBB regions and interact with endothelial cells, thus promoting permeability changes and inflammatory signalling [25]. Although the platelet miRNome remains markedly underexplored in the context of MS, circulating miRNAs have been the subject of sustained research over many years as prospective diagnostic biomarkers for the disease.

Our PCR validation confirmed that let-7a-5p was one of the most markedly downregulated miRNAs in platelets from MS patients. The available literature indicates that let-7a plays a regulatory role in microglial inflammatory responses and neuronal survival. Its downregulation promotes proinflammatory microglial activation, while increased expression favours an anti-inflammatory phenotype, reduces apoptosis and nitric oxide synthase (iNOS)-mediated nitric oxide production, and enhances BDNF expression. These neuroprotective effects may involve modulation of TLR/NF-κB and sirtuin 1 (SIRT1)-dependent signalling pathways [26]. A significant reduction in let-7i-5p expression was also detected in platelets from MS patients (Figure 5A, Table 3). Evidence from the literature suggests that the let-7 family contributes to immune homeostasis by regulating Th17 differentiation and migration, as demonstrated in the EAE, an animal MS model [27]. However, the findings regarding let-7i expression in MS remain inconsistent. Kimura et al. (2018) reported an increase in exosomal let-7i expression associated with impaired Treg induction [28], while a more recent study based on an extracellular vesicle identified a nonsignificant decreasing trend in let-7i-5p expression [29]. Taken together, let-7i seems to be a good candidate for further research, with documented involvement in the pathogenesis of MS, probably by regulating the dynamic balance of the Treg/Th17 axis critical for immune homeostasis.

MiR-15b-5p regulates growth factor-associated signaling pathways, including vascular endothelial growth factor A (VEGFA), fibroblast growth factor 2 (FGF-2), insulin-like growth factor 1 receptor (IGF1R), and the phosphoinositide 3-kinase/protein kinase B (PI3K/AKT) pathways, and has been implicated in platelet-derived growth factor (PDGF)-mediated vascular remodeling [30,31,32,33]. In our study, expression of miR-15b-5p in platelets from MS patients coincided with increased surface GPVI expression (Figure 3), enhanced platelet aggregation, P-selectin expression, and platelet-derived microparticle release (Figure 2A–C). Given the involvement of GPVI-mediated platelet activation in thrombo-inflammatory responses and vascular injury, the downregulation of miR-15b-5p may contribute to the prothrombotic platelet phenotype observed in MS by altering PDGF-dependent signaling and platelet-vascular interactions [34,35]. However, the proposed mechanistic relationship requires experimental validation.

Available evidence indicates that the biological activity of miR-126 arises from its coordinated regulation of multiple target proteins that govern angiogenesis and vascular integrity. In particular, miR-126 is highly expressed in platelets and endothelial cells and serves as a key regulator of vascular homeostasis [36]. Our demonstrated decreased expression of miR-126-3p in platelets in MS also supports the loss of control over the mechanisms of integrity of the vascular wall. Another miRNA involved in vascular hemostasis is miR-142-3p. Bao et al. (2018) demonstrated that PMP-mediated transfer of miR-142-3p induces aberrant endothelial proliferation through Bcl-2-associated transcriptional pathways, contributing to vascular dysfunction [37]. Although our studies showed reduced levels of platelet miR-142-3p, a marked increase in the percentage of PMPs generated by MS platelets may enhance horizontal transfer of miR-142-3p, thus amplifying endothelial changes. Our chosen miR-146a-5p is a well-known negative regulator of inflammation, acting through suppression of the IRAK1/TRAF6/NF-κB signalling axis. Its anti-inflammatory role has been widely confirmed in both cells of the innate immune system and platelets, where it limits excessive activation [38]. Therefore, the observed reduction in the expression of miR-146a-5p in platelets from MS patients may promote a pro-inflammatory phenotype, which is determined by enhanced NF-κB signalling and increased release of inflammatory mediators.

MiR-223 represents a key platelet miRNA with a well-established role in regulating platelet reactivity, primarily through modulation of P_2_Y_12_ receptor-dependent signalling pathways [39]. Its involvement in ADP-mediated activation and its incorporation into PMPs position miR-223 as a regulator and effector of platelet-driven inflammatory and thrombotic responses. Beyond platelets, miR-223 can be transferred to endothelial cells through PMPs, where it exerts post-transcriptional control over genes involved in vascular integrity and endothelial activation, linking platelet activation with vascular and immune dysregulation [40].

Our recent studies have focused on extracellular vesicle (EV)-derived miRNAs as potential minimally invasive biomarkers for disease stratification. Plasma-derived EVs isolated from patients with stable RRMS, SPMS, and healthy controls were profiled with RNA sequencing and validated by Real-Time PCR. This approach identified a distinct set of EV-associated miRNAs, including miR-760, miR-98-5p, miR-301a-3p, and miR-223-3p, that significantly differed between RRMS and SPMS phenotypes [41]. In the present study, we further demonstrate that miR-223 is selectively altered at the megakaryocyte level (Figure 5B, Table 3), indicating that its dysregulation may originate during thrombopoiesis and reflect disease-specific reprogramming of platelet precursors in MS.

Platelet-derived miRNAs profiling in MS remains unexplored in sufficient detail, despite increasing evidence supporting platelet transcriptomics and circulating miRNAs as biomarker sources. Previous studies demonstrated a high diagnostic performance of platelet RNA signatures in MS (AUC ~0.89), confirming the biological relevance of platelets in disease-related processes [42]. In parallel, other studies have applied ROC analysis to circulating miRNAs, indicating their utility in determining response to fingolimod in MS patients; however, these analyses were primarily based on plasma, serum, or peripheral blood mononuclear cells rather than platelets [43].

Although the two-miRNA model showed good discrimination during internal validation (AUC = 0.89; sensitivity, 89%; specificity, 85%), its performance decreased substantially in the external cohort (Harrell’s C-index = 0.66; sensitivity, 50%; specificity, 73%) (Figure 7), limiting its current diagnostic utility. Such reductions are frequently reported for clinical prediction models, including molecular classifiers [44,45]. In our study, the considerable variability in platelet counts among MS patients in the external cohort (121–623 × 10^3^/µL) may reflect biological heterogeneity relevant to miRNA expression. Differences in other patient characteristics may also have contributed to the reduced performance. Another possible explanation is overfitting in the development cohort. However, the final logistic regression model included only two predictors, corresponding to 13 events per predictor, meeting the conventional threshold of 10 [46]. Nevertheless, this criterion and the use of 10-fold cross-validation cannot fully exclude overfitting, particularly when predictors are selected from a larger set of candidates [47]. Importantly, independent external validation and transparent reporting of its results represent a strength of our study, providing a more realistic assessment of the model’s generalizability. Further optimization and validation in larger, more heterogeneous cohorts are therefore required before clinical application. Logistic regression modelling with miRNAs therefore represents a novel approach. Given the active role of platelets in neuroinflammation and BBB interactions, their miRNA cargo may provide disease-specific signals that are not captured by other biofluids. This supports the rationale for using platelet let-7a and miR-146a-5p as candidates for MS biomarkers.

Exploratory analyses using KEGG and miRDB identified potential molecular pathways and regulatory interactions associated with the miRNAs investigated in our study.

KEGG pathway enrichment analysis (Figure 8) suggested potential associations with neurodegenerative processes, including mitochondrial dysfunction, oxidative stress, and impaired proteostasis, as well as MAPK, mTOR, and inflammatory signalling pathways [48,49,50,51,52,53,54,55]. Complementary miRDB analysis (Table 6) identified shared predicted miRNA targets involved in miRNA biogenesis (*DICER1*, *AGO1*, *AGO4*), intracellular signaling (*PI3K/AKT*, *cAMP/PKA*, *TGF-β*), cytoskeletal organization, vesicular transport, and circadian regulation (*CLOCK*) [56,57,58,59].

Our in silico analysis revealed that let-7a-5p and miR-146a-5p are the only two miRNAs with high potential to discriminate MS from healthy controls, as demonstrated by logistic regression analysis. In MS, platelet-derived let-7a and miR-146a-5p were consistently negatively regulated and did not show significant intercorrelation (Figure 7, Table 4), indicating their independent contribution to disease-associated molecular alterations and supporting their joint inclusion as complementary biomarkers. A bivariate logistic model incorporating both miRNAs demonstrated strong discriminative capacity in the discovery cohort, explaining a substantial proportion of variance and achieving high sensitivity and specificity upon internal validation (Table 4).

Next, computational PPI network analysis of the predicted let-7a-5p and miR-146a-5p target genes (Figure 9, Table 6) identified potential protein interaction networks associated with key pathways relevant to MS pathophysiology. The PPI network included proteins associated with immune regulation (*IL6*, *RORC*, *IRAK1*), BBB integrity and vascular function (*COL4A1*, *PDGFRB*), myelination (*GALC*, *LINGO1*), oxidative stress (*NOX4*, *GPX6*), synaptic transmission (GRIA3, SYT1), and epigenetic regulation (*EHMT2*) (Figure 9, Table 6). These associations suggest potential molecular pathways for future investigation rather than confirmed mechanisms of MS pathophysiology. All proposed associations between the analyzed miRNAs, the predicted target genes, and the pathological processes related to MS remain hypothetical. Therefore, these findings should be considered hypothesized and may help prioritize molecular targets for future experimental validation.

Nevertheless, the present study has certain limitations that need to be addressed in future research. Despite the inclusion of an independent external validation cohort, the reduced sensitivity observed during validation highlights the need for more studies in larger and more diverse populations. Moreover, the cross-sectional design precludes the assessment of temporal changes in miRNA expression during MS progression. Given the limited disease specificity of platelet-associated miRNAs, prospective longitudinal studies are warranted to establish their potential clinical utility as MS biomarkers. Furthermore, it remains unclear at what stage of MS these alterations emerge and how they evolve throughout the course of the disease, highlighting the need for longitudinal studies involving different MS phenotypes to establish their potential clinical relevance. It should also be noted that computational approaches facilitate the identification of potential miRNA regulatory networks and candidate biomarkers by predicting molecular interactions and disease-associated pathways [60]; however, their reliability is limited by database quality, false-positive predictions, and insufficient cell-specific information. In our study, the predicted interactions remain hypothetical and do not confirm functional gene regulation or the transfer of platelet-derived miRNAs to recipient cells. Therefore, these findings should be considered hypothesis-generating and require independent experimental clinical validation to establish their biological relevance and potential biomarker utility in MS.

## 4. Materials and Methods

### 4.1. Flow Cytometry

The levels of specific markers of platelet activation, including platelet aggregate (PAG) formation, platelet microparticles (PMPs), and P-selectin (CD62P), were evaluated using flow cytometry, as previously described [9,10,11]. All blood samples were processed within 30 min of collection. Briefly, fresh whole blood samples, both untreated and activated with Chrono-Par^®^ Collagen (at final concentration 1 µg/mL, 10 min, 37 °C) (Chrono-log Corp.Havertown, PA, USA), were labelled with mouse anti-human CD61/FITC (clone RUU-PL7F12) alone or in combination with anti-human CD62P/PE (clone AC1.2) antibodies (Becton Dickinson, Franklin Lakes, NJ, USA) for 30 min in the dark at room temperature (RT), followed by fixation in 1% CellFIX solution (Becton Dickinson, Franklin Lakes, NJ, USA) for 15 min at RT. All samples from patients with MS and healthy volunteers were processed using the same standardized staining, fixation, and acquisition procedures to minimize preanalytical and analytical variability between groups. Gates for PE and FITC fluorescence were set based on the fluorescence of unstained controls. Fluorescence-minus-one (FMO) controls were used to establish the boundary between positive and negative platelets. CD61-positive objects with a forward scatter (FSC) parameter on a log scale above 10^3.5^ were classified as PAGs, while those below 10^2.2^ were classified as PMPs. Surface expression of P-selectin on CD61/FITC-positive cells was assessed using the fluorescence of CD62/PE. The percentage of CD61+CD62P+ platelets was calculated relative to the total platelet population in each sample (15,000 CD61+ events). The flow cytometric gating strategy, including representative histograms illustrating the identification and characterization of platelet populations, is provided in the Supplementary Figure S2. The compensation procedure was performed using the VersaComp Antibody Capture Bead Kit (Beckman Coulter, Brea, CA, USA) as recommended by the manufacturer. Analyses were performed using Cube 6 Flow Cytometry (Sysmex Partec, Görlitz, Germany) with CyView software v.1.5.5.8 (Sysmex Partec, Görlitz, Germany).

### 4.2. Blood Platelet Isolation and Leukocyte/Erythrocyte Depletion

Platelet-rich plasma (PRP) was obtained by collecting fresh whole blood samples from subjects using an S-Monovette^®^ CPDA-1 (Sarstedt, Nümbrecht, Germany) system, followed by immediate centrifugation at 235× *g* for 12 min at RT as previously described [9,10,11]. The top layer of PRP was carefully collected and transferred to a fresh tube. To prevent spontaneous platelet activation during processing, prostaglandin E1 (PGE1) (Sigma-Aldrich, St. Louis, MO, USA) was added to achieve a final concentration of 300 nM. To remove erythrocyte and leukocyte contamination, PRP was purified using nano-sized MicroBeads conjugated with monoclonal human antibodies specific to CD235a (erythrocyte marker) and CD45 (leukocyte marker) (Miltenyi Biotec, Bergisch Gladbach, Germany). The efficiency of erythrocyte/leukocyte depletion was confirmed using flow cytometry, employing anti-CD235a/FITC-conjugated (clone GA-R2 (HIR2)) and anti-CD45/PE-conjugated antibodies (clone HI30) to target platelet and leukocyte surface markers, respectively. Microscopic analysis further confirmed the absence of erythrocyte/leukocyte contamination, consistent with flow cytometry findings. Platelet concentration was spectrophotometrically determined and adjusted to a final concentration of 2 × 10^8^ platelets/mL using modified Tyrode’s buffer (pH 7.4) containing 134 mM NaCl, 12 mM NaHCO_3_, 2.9 mM KCl, 0.4 mM Na_2_HPO_4_·12H_2_O, 1 mM MgCl_2_·6H_2_O, 10 mM HEPES, 5 mM glucose, and deionized water (all from Sigma-Aldrich). Finally, blood platelet suspension was divided into two equal portions; in one part, RNAlater^TM^ stabilisation (Thermo Fisher Scientific) was added, and in the second part, cell lysis buffer (Thermo Fisher Scientific, Waltham, MA, USA) was added. Then they were immediately frozen (−80 °C) for further transcriptomic and proteomic analysis.

### 4.3. Transmission Electron Microscopy (TEM)

Based on our previously described protocol [11], blood platelet samples were fixed immediately after isolation, within 30 min of blood collection, in 2.5% glutaraldehyde in 0.1 M PBS (pH 7.4) for 3 h at 4 °C, centrifuged (1743× *g*, 10 min), and embedded in 2% agar. After PBS rinsing, samples were postfixed in 1% osmium tetroxide (2 h, 4 °C), dehydrated in graded ethanol, and stained overnight with uranyl acetate in 50% ethanol. Following dehydration and propylene oxide treatment, samples were embedded in Epon–Spurr resin and polymerized (37–72 °C). The same standardized isolation and fixation procedures were consistently applied to all samples from patients with MS and healthy volunteers to minimize preanalytical variability and potential ex vivo platelet activation. All reagents were purchased from Polysciences, Inc. (Warrington, PA). Ultrathin sections (60–70 nm) were prepared using an Ultra Cut E ultramicrotome (Reichert Jung, Germany, Vienna, Austria), contrasted with uranyl acetate and lead citrate, and examined with a JEM 1010 transmission electron microscope (JEOL Ltd., Tokyo, Japan) at 80 kV. Images were digitized using a Perfection V700 PHOTO scanner (Epson, Suwa, Japan).

### 4.4. Megakaryocyte Isolation

Megakaryocytes were isolated from whole blood through a two-stage process as previously described [9,10]. Initially, peripheral blood mononuclear cells (PBMCs) were separated by gradient centrifugation. Fresh whole blood was layered onto Histopaque^®^-1077 (Sigma-Aldrich, St. Louis, MO, USA) in a 1:1 ratio and centrifuged at 635× *g* for 30 min at RT. The resulting PBMC fraction was carefully collected, washed twice with PBS (pH 7.4) (Sigma-Aldrich), and centrifuged at 400× *g* for 12 min at RT. The PBMC pellet was resuspended in reaction buffer (pH 7.4) containing 2 mM ethylenediaminetetraacetic acid (EDTA) (Sigma-Aldrich, St. Louis, MO, USA) and 0.5% bovine serum albumin (BSA) (Sigma-Aldrich, St. Louis, MO, USA). In the second stage, CD61-positive megakaryocytes were magnetically labeled using anti-CD61 microbeads (Miltenyi Biotec, Bergisch Gladbach, Germany) and isolated from the PBMC suspension via magnetic separation. The cell suspension was applied to a MACS^®^ column under the influence of a magnetic field, which captured the CD61-positive cells while allowing CD61-negative cells to flow through. The positively selected megakaryocyte fraction was eluted with reaction buffer by applying negative pressure with the column plunger. Megakaryocyte numbers were quantified using a Cube6 flow cytometer (Sysmex Partec GmbH, Görlitz, Germany) by labeling with anti-CD61/FITC-conjugated (clone RUU-PL7F12) and anti-CD42/PE-conjugated (clone HIP1) antibodies (Becton Dickinson, Franklin Lakes, NJ, USA). The purity of the megakaryocyte suspension was assessed cytometrically using antibodies against leukocyte/erythrocyte antigens: anti-CD45/PE-conjugated (clone HI30) and anti-CD235a/FITC-conjugated (clone GA-R2/HIR2) (Becton Dickinson, Franklin Lakes, NJ, USA). Finally, megakaryocyte suspension was divided into two equal portions; in one part, the RNAlater^TM^ stabilization (Thermo Fisher Scientific, Waltham, MA, USA) was added, and in the second part, the cell lysis buffer (Thermo Fisher Scientific, Waltham, MA, USA) was added. Then, they were immediately frozen (−80 °C) for further transcriptomic and proteomic analysis.

### 4.5. Enzyme-Linked Immunosorbent Assay (ELISA)

First, the total protein concentration was determined using the RC DC™ Protein Assay (Bio-Rad, Hercules, CA, USA) method with a bovine serum albumin (BSA) standard curve. Each sample was adjusted to a uniform concentration, specifically 2 μg/μL for blood platelets and 1 μg/μL for megakaryocytes. The expression level of the GPVI receptor in blood platelets and megakaryocytes was quantified using a commercial Human GP6 (Glycoprotein VI Platelet) ELISA kit (Elabscience, Wuhan, China), according to the manufacturer’s instructions. Briefly, standards and samples (100 μL) were added to the wells and incubated for 90 min at 37 °C. After removing the liquid, 100 μL of biotinylated detection antibody was added and incubated for 1 h at 37 °C. The wells were washed three times and then incubated with 100 μL of HRP (horseradish peroxidase) conjugate for 30 min at 37 °C. Following five washing cycles, 90 μL of substrate solution was added and incubated for 15 min at 37 °C. Absorbance was recorded at 450 nm using a SPECTROstar Nano microplate reader (BMG Labtech, Ortenberg, Germany). GPVI concentrations were calculated from a standard calibration curve and expressed as ng/mL.

### 4.6. RNA Isolation and RT-PCR

Before RNA isolation, a spike-in control *cel*-miR-39 (Norgen Biotek Corp., Thorold, ON, Canada) was added at a final concentration of 10 pM. Total RNA extraction from frozen (at −80 °C) blood platelets and megakaryocytes suspended in RNAlater^TM^ solution was performed using the ISOLATE II RNA Mini Kit (Bioline, London, UK), following the manufacturer’s instructions. RNA purity was assessed using a spectrophotometer Synergy HIT on the Take3™ microvolume plate (Agilent Technologies, Santa Clara, CA, USA); RNA integrity was examined on TapeStation 2200 using High-Sensitivity RNA ScreenTape (Agilent Technologies, Santa Clara, CA, USA), while concentration was measured using a Qubit 3.0 Fluorometer (Thermo Fisher Scientific, Waltham, MA, USA). All total RNA samples were stored at −80 °C until further use. For reverse transcription PCR (RT-PCR), the Maxima First Strand cDNA Synthesis Kit (Thermo Fisher Scientific, Waltham, MA, USA) was employed according to the manufacturer’s protocol. The resulting cDNA libraries were also stored at −80 °C until they were analysed by Real-Time PCR.

### 4.7. Microarray Analysis

Microarray analysis utilised the Microarray SurePrint G3 Human miRNA r21 Array Kit (Agilent Technologies, Santa Clara, CA, USA), designed for high-throughput processing with optimal sequence discrimination and sensitivity. The microarray slides featured 2549 human miRNAs sourced from miRBase Database Release 21.0, employing SurePrint inkjet technology to synthesise 40- to 60-mer oligonucleotides directly on the slide in an array format. Approximately 15,000 printed probes were distributed randomly across the slide to ensure complete coverage of all miRNAs in the tested samples. Total RNA samples stored at −80 °C were transported on dry ice to the Institute of Biology of Jan Kochanowski University in Kielce, Poland, for processing. The experimental protocol included several critical steps: preparation of spike-in solutions for the generation of fluorescently labelled miRNA, and sample dephosphorylation, denaturation, ligation, and drying procedures. Following these steps, microarray slides were placed in a SureHyb chamber cover and incubated in an oven rotated at 55 °C for 24 h at 20 rpm. After hybridisation, the slides were washed and scanned. To prevent post-wash complications, slides were immediately placed in ozone-barrier slide covers. Scanning was conducted using the SureScan Agilent G2565C scanner with Feature Extraction software v. 10.10.1.1 (Agilent Technologies, Santa Clara, CA, USA), utilising grid number 070156_D_F_20141006.

### 4.8. Real-Time PCR

The validation analysis of the gene expression for ten selected miRNA molecules was made using TaqMan^®^ probes (Life Technologies, Carlsbad, CA, USA) (Table 7). Quantitative data were normalized to the level of cel-miR-39-3p (exogenous control) and 191-5p (endogenous control). The selection of miR-191-5p as an endogenous reference was supported by previous studies that used this miRNA as an internal control in human platelet samples, including research involving patients with acute coronary syndrome [61] and investigations of miRNA expression in stored human platelets [62]. To account for both endogenous miRNA abundance and technical variability, *cel*-miR-39-3p was also included as an exogenous spike-in control. The combined reference Ct was calculated as the median of the Ct values obtained for miR-191-5p and cel-miR-39-3p and subsequently used for normalisation of target miRNA expression. Real-time PCR was performed in a CFX96™ Real-Time PCR Detection System Thermal Cycler (Bio-Rad, Hercules, CA, USA) using a TaqMan Universal Master Mix II, no UNG (Life Technologies, Carlsbad, CA, USA). All steps were performed according to the manufacturer’s protocol, including an initial polymerase activation at 95 °C for 20 s, followed by 40 cycles of denaturation at 95 °C for 3 s and extension at 60 °C for 30 s. Equation −ΔCt (ΔCt = Ct target gene—Ct means of two control genes) was used to calculate the relative expression of studied miRNAs.

### 4.9. Data Analysis

Real-Time PCR data that were missing, likely in a random pattern (two cases for platelets and four cases for megakaryocytes), were imputed using the multiple imputation by chained equations method using R software v. 4.4.1 with package mice v. 3.17.0 (R Foundation for Statistical Computing, Vienna, Austria). Microarray data analysis was performed using GeneSpring GX software v. 14.9.1; (Agilent Technologies, Santa Clara, CA, USA), following the manufacturer’s protocol throughout all stages of the experiment. Real-Time PCR data were presented and treated as −ΔCt to keep the distribution close to normal (the higher the value, the higher the miRNA expression), and FC was reported. Descriptive statistics included mean with standard deviation for continuous variables, median with 1st (Q1) and 3rd (Q3) quartiles for ordinal variables, and number with frequency for categorical variables. The differences between two means of variables with distributions close to normal were tested with Student’s *t*-test, while variables that were highly skewed were tested with the Mann–Whitney U test. Benjamini and Hocheberg (B-H) correction for multiple testing was applied for microarray analysis to control the false discovery rate at 0.05. For covariate-adjusted fold changes in miRNA expression, sex, age, BMI, and platelet count were used linearly linked to the outcome with the use of general linear modelling. Exploratory factor analysis (EFA) of residualised values was used to examine intercorrelations among miRNAs tested. Factors were extracted using the principal component method with orthogonal varimax rotation. Pearson’s r and Spearman’s rho, together with exploration factor analysis, were used to present correlation matrices and examine the collinearity of miRNA and platelet parameters. Logistic regression was used to evaluate the discriminative properties of selected miRNA between MS patients and controls. To assess the goodness of fit, the Hosmer–Lemeshow chi-squared test was performed. The receiver operating characteristic (ROC) curve analysis was applied to evaluate model performance in the total cohort and after internal validation using 10-fold cross-validation. Areas under the ROC curves (AUCs) with 95% confidence intervals (CIs) were estimated. A cut-off point was proposed based on the maximization of Youden’s index. Harrell’s C-index, together with sensitivity and specificity measures, was reported for external validation of the predictive model. KEGG pathway enrichment analyses were performed to determine the biological significance of associated proteins, while a bubble plot was generated using RStudio v. 4.6.0 (Boston, MA, USA). In each analysis, *p*-values below 0.05 were considered statistically significant. The analysis was performed using STATISTICA v. 13.3 software(StatSoft, Tulsa, OK, US). Graphs and plots were prepared using GraphPad software v. 8.0.1 (GraphPad Inc., San Diego, CA, USA), MS Excel (Microsoft Corporation, Redmond, WA, USA), or STATISTICA v. 13.3 (StatSoft, Tulsa, OK, USA), while STRING v. 12.5 ( https://string-db.org accessed on 20 May 2026) was used to generate protein–protein interactions (PPIs).

## 5. Conclusions

This study identifies platelet-derived miRNAs as a previously unrecognized mechanistically relevant layer of MS biology. Coordinated downregulation of key regulatory miRNAs, particularly let-7a-5p and miR-146a-5p, may have functional relevance in the pathophysiology of MS. In silico analyses demonstrated that these miRNAs are linked to numerous target genes involved in several biological pathways that are central to the development and progression of MS, including neuroinflammation, BBB dysfunction, immune regulation, and neurodegenerative processes. Although the two-miRNA signature showed good discriminatory performance during internal validation, its reduced performance in the external cohort, particularly its low sensitivity, indicates that further optimization and validation are required before clinical application. Together, these findings position platelets as active amplifiers of MS pathophysiology and a source of robust, functionally informative biomarkers.

## 6. Patents

Blood samples from MS patients and control subjects were collected in Sarstedt^®^ citrate phosphate dextrose adenine-1 tubes (CPDA-1) (Nümbrecht, Germany) under fasting conditions in the morning hours. The samples were stored under identical conditions following a standardized protocol. The clinical and anthropometric characteristics of the control group and MS patients are presented in Table 8.

The study included 26 MS patients (18 females and 8 males) with an age of 52 ± 12 years. The patients were diagnosed with MS according to the revised McDonald criteria and using magnetic resonance imaging (MRI). The secondary progressive (SP) course of MS (inactive with progression) was defined according to Lublin et al. [63]. MS patients did not receive any immunomodulators or hormones. Participants were enrolled in the study after completing a medical questionnaire and providing consent. The inclusion criteria included individuals aged 40 to 65 years, with an Expanded Disability Status Scale (EDSS) score ranging from 4 to 7, a modified Rankin scale (mRS) score ranging from 2 to 4, and a disease duration of 15–30 years.

Exclusion criteria included treatment with medications known to affect platelet count, activation, or function, including antiplatelet agents, nonsteroidal anti-inflammatory drugs (NSAIDs), selective serotonin reuptake inhibitors (SSRIs), serotonin–norepinephrine reuptake inhibitors (SNRIs), anticoagulants, and systemic corticosteroids. Other exclusion criteria comprised neurological comorbidities, depression or other psychiatric disorders, those receiving disease-modifying therapy (DMT) (i.e., interferon-β, glatiramer acetate, or natalizumab), immunomodulatory or immunostimulatory treatment, and recent infection within four weeks before blood collection.

Control blood samples were collected from 26 healthy volunteers at the Laboratory Diagnostics Centre in Lodz, Poland. The control group was matched by age (47 ± 13) and gender (14 females and 12 males). In the control group, acute or chronic comorbidities were not allowed. Consequently, control subjects had no history or clinical evidence of neurological, autoimmune, inflammatory, hormonal, hematologic, cardiovascular, or coagulation disorders and did not receive any pharmacological treatment or dietary supplementation affecting platelet or immune function. Each volunteer in the control group was tested, including hematological and biochemical tests, to confirm their health status.

**Table 8:** Clinical characteristics of the control group and MS patients.

Parameters	Control Group ^a^ (*n* = 26)	MS ^a^ (*n* = 26)	Difference ^b^
Age [years]	47.2 ± 13.1	52.0 ± 12.0	t = 1.37 (df = 50) *p* = 0.18
Gender [F—female; M—male]	F: 14 (54%) M: 12 (46%)	F: 18 (69%) M: 8 (31%)	χ^2^ = 1.30 (df = 1) *p* = 0.25
Body mass index (BMI) [kg/m^2^] 15–19.9: Underweight 20–24.9: Normal 25–29.9: Overweight 30: Obesity [64]	24.2 ± 2.0	26.1 ± 2.7	t = 2.77 (df = 50) *p* = 0.0078 *
Number of blood platelets [× 10^3^/μL]	285 ± 63	304 ± 68	t = 1.04 (df = 50) *p* = 0.31
Mean duration of the disease (MDD) [years]	N/A	22.5 ± 6.0	-
Expanded Disability Status Scale (EDSS) 0: No disability 1–2.5: Minimal disability 3–3.5: Moderate disability 4–4.5: Significant disability 5–5.5: Severe disability 6–6.5: Requires a walking aid 7–7.5: Unable to walk 8–9.5: Confined to bed 10: Death [65,66]	N/A	6 (6–6.5)	-
Modified Rankin Scale (mRS) 0–1: No significant disability 2–3: Moderate disability 4–5: Severe disability 6: Death [67]	N/A	3 (3–3)	-
Beck’s Depression Inventory (BDI) 0–9: Minimal depression 10–18: Mild depression 19–29: Moderate depression 30: Severe depression [68]	N/A	14 (12–15)	-
Mini-Mental State Examination (MMSE) 27: No cognitive impairment 21–26: Mild impairment 11–20: Moderate impairment <10: Severe impairment [69]	N/A	25 (23–27)	-
Montreal Cognitive Assessment (MoCA) 26–30: Normal 18–25: Mild cognitive impairment 10–17: Moderate cognitive impairment <10: Severe cognitive impairment [70]	N/A	26.5 (25–29)	-
Activity of Daily Living (ADL) The scale evaluates the basic activities of daily living of a patient. Each activity is scored from 5 points (able to perform the activity independently and without difficulty) to 1 point (unable to perform the activity) [71]	N/A	13 (11–14)	-

Legend: ^a^ descriptive statistic: mean ± standard deviation (SD) OR median (with the first and third quartiles Q1 and Q3) OR number (frequency, %); ^b^ statistical test and *p*-value; df—degrees of freedom; N/A—not applicable. * Although BMI differences were observed between groups, none of the participants presented obesity-associated metabolic or inflammatory disorders that could confound platelet miRNA profiling.

To assess model robustness under biologically heterogeneous conditions, an independent external validation cohort consisting of 22 MS patients and 22 control subjects (Table 9), selected according to the same inclusion and exclusion criteria but not included in the initial model development, was used exclusively for external validation of the classification model. The MS subgroup within this cohort exhibited substantially greater variability in platelet counts than the primary cohort; therefore, a corresponding external control subset was also included to enable comparisons between patients with MS and healthy individuals.

## Figures and Tables

**Table 1:** Flow cytometric assessment of platelet activation and collagen reactivity in MS patients and controls.

Variant	Cytometric Measurements	Control Group ^a^ ( * n * = 26)	MS ^a^ ( * n * = 26)	Difference ^b^
Non-stimulated	Platelet aggregates [%]	6.6 (4.6–7.9)	12.8 (11.9–15.1)	Z = 5.72, *p* < 0.0001
	Platelet-derived microparticles [%]	4.0 (3.1–6.4)	12.4 (8.8–17.0)	Z = 5.76, *p* < 0.0001
	P-selectin–positive platelets [%]	0.5 (0.3–0.7)	6.2 (4.6–8.5)	Z = 6.18, *p* < 0.0001
Collagen-stimulated	Platelet aggregates [%]	10.9 (8.9–14.6)	22.4 (17.6–27.1)	Z = 5.70, *p* < 0.0001
	Platelet-derived microparticles [%]	5.8 (3.7–7.7)	11.3 (8.8–12.8)	Z = 5.90, *p* < 0.0001
	P-selectin–positive platelets [%]	19.7 (15.2–23.9)	24.0 (19.0–31.5)	Z = 2.33, *p* = 0.019

Legend: ^a^ median (with Q1 and Q3); ^b^ Z-test and *p*-value (Mann–Whitney U test).

**Table 2:** Differentially expressed miRNAs selected for Real-Time PCR validation based on platelet and megakaryocyte microarray profiling. Blue downward arrows (↓) indicate downregulated miRNAs, whereas red upward arrows (↑) indicate upregulated miRNAs relative to the control group.

miRNA	*p*-Value	*p*-Value ^a^ (B-H Adjusted)	Log FC ^b^ (Regulation)	*p*-Value	*p*-Value ^a^ (B-H Adjusted)	Log FC ^b^ (Regulation)
	Blood Platelets			Megakaryocytes		
let-7a-5p	0.0011	0.0033	−4.32 (↓)	0.0014	0.0022	−1.17 (↓)
let-7i-5p	0.0066	0.0084	−1.43 (↓)	0.0029	0.0040	−1.07 (↓)
15b-5p	0.0003	0.0022	−5.31 (↓)	0.0000	0.00001	2.12 (↑)
16-5p	0.0005	0.0025	−5.88 (↓)	0.0000	0.0000	2.21 (↑)
21-5p	0.0000	0.0005	−3.37 (↓)	0.0000	0.0000	4.13 (↑)
107	0.0006	0.0025	−4.57 (↓)	0.0000	0.0000	4.25 (↑)
126-3p	0.0009	0.0028	−5.13 (↓)	0.0000	0.0000	5.61 (↑)
142-3p	0.0014	0.0034	−1.96 (↓)	0.0002	0.0004	1.72 (↑)
146a-5p	0.0023	0.0042	−2.51 (↓)	0.0000	0.0000	3.26 (↑)
223-3p	0.0006	0.0025	−4.39 (↓)	0.0000	0.0000	2.23 (↑)

Legend: ^a^ Moderated T test with Benjamini–Hochberg (B-H) correction. ^b^ Raw microarray data were background-corrected and normalized using percentile shift normalization (75th percentile) in GeneSpring software (version 14.9.1; Agilent Technologies, Santa Clara, CA, USA), followed by log2 transformation.

**Table 3:** Expression profiles of platelet- and megakaryocyte-selected 10 miRNAs in MS patients and controls measured by Real-Time PCR.

miRNA		Control Group (*n* = 26)		MS (*n* = 26)		FC (Raw)		FC (Covariate-Adjusted Analysis)	
		Mean −ΔCt	SD	Mean −ΔCt	SD	Point Estimation (95% CI)	*p*-Value	Point Estimation (95% CI) ^a^	*p*-Value ^a^
Blood platelets	let-7a-5p	−0.54	2.86	−4.77	3.16	0.053 (0.017–0.170)	<0.0001	0.064 (0.019–0.213)	0.0001
	let-7i-5p	−0.02	2.14	−4.03	3.47	0.062 (0.020–0.189)	<0.0001	0.071 (0.022–0.232)	0.0001
	15b-5p	−2.04	1.24	−5.68	3.38	0.080 (0.030–0.214)	<0.0001	0.090 (0.030–0.267)	0.0001
	16-5p	−1.26	1.33	−3.23	3.37	0.255 (0.095–0.686)	0.0078	0.411 (0.147–1.15)	0.11
	21-5p	−2.27	1.07	−5.26	3.98	0.126 (0.041–0.388)	0.0005	0.157 (0.045–0.552)	0.0081
	107	−5.13	1.47	−8.67	4.01	0.086 (0.027–0.276)	0.0001	0.106 (0.030–0.373)	0.0016
	126-3p	−2.01	1.30	−5.51	4.21	0.088 (0.027–0.295)	0.0002	0.098 (0.027–0.359)	0.0016
	142-3p	−2.00	1.37	−5.77	5.37	0.073 (0.016–0.333)	0.0011	0.133 (0.026–0.679)	0.025
	146a-5p	−2.24	1.19	−6.46	3.77	0.054 (0.018–0.158)	<0.0001	0.057 (0.017–0.188)	<0.0001
	223-3p	−0.26	1.45	−0.33	1.81	0.952 (0.506–1.792)	0.88	0.859 (0.426–1.73)	0.69
Megakaryocytes	let-7a-5p	−2.48	2.41	−3.34	2.24	0.548 (0.223–1.35)	0.18	0.787 (0.303–2.04)	0.64
	let-7i-5p	−2.08	2.27	−1.30	2.13	1.72 (0.733–4.02)	0.21	2.02 (0.810–5.06)	0.15
	15b-5p	−5.22	6.96	−3.15	5.16	4.19 (0.393–44.7)	0.23	2.40 0.188–30.6)	0.52
	16-5p	−3.18	2.18	−2.27	1.97	1.87 (0.837–4.17)	0.12	1.40 (0.603–3.24)	0.46
	21-5p	−6.15	5.94	−4.55	5.52	3.03 (0.331–27.7)	0.32	1.245 (0.113–13.7)	0.86
	107	−8.00	4.03	−7.13	2.90	1.84 (0.473–7.12)	0.37	2.78 (0.683–11.3)	0.18
	126-3p	−8.04	6.08	−5.17	3.44	7.29 (1.08–49.1)	0.04	5.96 (0.772–46.1)	0.11
	142-3p	−4.76	3.38	−4.31	4.61	1.37 (0.287–6.53)	0.69	0.567 (0.109–2.94)	0.52
	146a-5p	−5.36	3.43	−4.41	2.72	1.93 (0.583–6.37)	0.28	1.96 (0.532–7.22)	0.34
	223-3p	−4.17	3.33	−0.53	2.17	12.5 (4.22–37.0)	<0.0001	9.97 (3.06–32.5)	0.0007

Legend: ^a^ Adjusted for sex, age, BMI, and platelet count.

**Table 4:** Multivariable logistic regression analysis of platelet let-7a-5p and miR-146a-5p expression in MS patients.

Variable	OR (95%CI)	Wald Statistics	*p*-Value
let-7a-5p	0.653 (0.467–0.914)	6.19	0.013
miR-146a-5p	0.573 (0.379–0.866)	6.97	0.0083

Hosmer–Lemeshow χ^2^(8) = 5.22, *p* = 0.73.

**Table 5:** Overlapping mRNA targets of ≥5 miRNAs validated by Real-Time PCR and annotated using miRDB.

Gene Symbol (mRNA Targets)	Full Gene Name (NCBI Gene ID)	Encoded Protein	miRNAs (≥5)
*DICER1*	Dicer 1, RNase III (Gene ID: 23405)	Endoribonuclease III	let-7a, let-7i, miR-15b, miR-16, miR-21
*AGO1*	Argonaute RISC Component 1 (Gene ID: 26523)	Argonaute protein 1	let-7a, let-7i, miR-15b, miR-16, miR-107
*AGO4*	Argonaute RISC Component 4 (Gene ID: 192669)	Argonaute protein 4	let-7a, let-7i, miR-15b, miR-16, miR-107
*HMGA1*	High Mobility Group AT-Hook 1 (Gene ID: 3159)	Chromatin-binding protein	let-7a, let-7i, miR-21, miR-107, miR-146a
*HMGA2*	High Mobility Group AT-Hook 2 (Gene ID: 8091)	Chromatin-binding protein	let-7a, let-7i, miR-21, miR-107, miR-146a
*POU2F1 (OCT1)*	POU Class 2 Homeobox 1 (Gene ID: 5451)	Transcription factor Oct1	let-7a, let-7i, miR-16, miR-21, miR-107
*ELK4*	ETS Transcription Factor ELK4 (Gene ID: 2005)	ETS-domain TF	let-7a, let-7i, miR-15b, miR-16, miR-107
*PIK3R1*	Phosphoinositide-3-Kinase Regulatory Subunit 1 (Gene ID: 5295)	PI3K p85α	let-7a, miR-21, miR-107, miR-126, miR-146a
*PRKAR2A*	Protein Kinase cAMP-Dependent Type II Regulatory Subunit Alpha (Gene ID: 5576)	PKA regulatory subunit	let-7a, let-7i, miR-16, miR-21, miR-107
*ACVR2A*	Activin A Receptor Type 2A (Gene ID: 92)	Ser/Thr kinase receptor	let-7a, let-7i, miR-15b, miR-16, miR-21
*ABL2 (ARG)*	Abelson Tyrosine Kinase 2 (Gene ID: 27)	Non-receptor tyrosine kinase	let-7a, let-7i, miR-21, miR-107, miR-146a
*ARHGAP12*	Rho GTPase Activating Protein 12 (Gene ID: 94134)	RhoGAP protein	let-7a, let-7i, miR-15b, miR-16, miR-107
*ARMC8*	Armadillo Repeat Containing 8 (Gene ID: 55154)	Adaptor protein	let-7a, let-7i, miR-16, miR-21, miR-107
*AMOT*	Angiomotin (Gene ID: 154796)	Scaffold protein	let-7a, let-7i, miR-15b, miR-16, miR-107
*CDC25A*	Cell Division Cycle 25A (Gene ID: 993)	Tyrosine phosphatase	let-7a, let-7i, miR-15b, miR-16, miR-21
*BTG2*	BTG Anti-Proliferation Factor 2 (Gene ID: 7832)	Anti-proliferative protein	let-7a, let-7i, miR-15b, miR-16, miR-107
*CLOCK*	Circadian Locomotor Output Cycles Protein Kaput (Gene ID: 9575)	Circadian transcription factor	let-7a, let-7i, miR-21, miR-146a, miR-223
*KPNA1*	Karyopherin Subunit Alpha 1 (Gene ID: 3836)	Nuclear import receptor	let-7a, let-7i, miR-16, miR-21, miR-107
*MOB4*	MOB Kinase Activator 4 (Gene ID: 79760)	Scaffold protein (Hippo pathway)	let-7a, let-7i, miR-16, miR-21, miR-107
*NAA30*	N-Alpha-Acetyltransferase 30 (Gene ID: 79685)	Acetyltransferase	let-7a, let-7i, miR-16, miR-21, miR-107

**Table 6:** Predicted target genes of let-7a-5p and miR-146a-5p and their functional relevance in MS pathophysiology using STRING database (https://string-db.org on 30 May 2026).

Gene Symbol (Gene ID Ensembl)	Encoded Protein	Molecular Function	Potential Link to MS Pathogenesis
*CACNA2D1* (ENSG00000153956)	Voltage-dependent calcium channel alpha-2/delta-1 subunit	modulate voltage-gated calcium channel activity; regulate excitability	neuronal excitability
*CCR7* (ENSG00000126353)	C-C chemokine receptor type 7	bind macrophage inflammatory protein 3 beta (CCL19); regulate lymphocyte migration	lymphocyte migration to CNS
*CD200R1* (ENSG00000163606)	Cell surface glycoprotein CD200 receptor 1	inhibit pro-inflammatory signalling; suppress immune activation;	microglia inhibition
*CLDN11* (ENSG00000163347)	Claudin-11	major role in tight junction formation; calcium-independent cell adhesion	BBB integrity, myelin sheath
*COL4A1* (ENSG00000187498)	Collagen IV alpha1	maintain the integrity of the basement membrane	BBB integrity
*COL4A2* (ENSG00000116016)	Collagen alpha-2(IV) chain	form the basement membrane structure; maintain the integrity of the barrier	BBB structure
*EHMT2* (ENSG00000106462)	Ubiquitin carboxyl-terminal hydrolase 3	methylate histone H3 lysine 9 (H3K9); regulate epigenetic repression	epigenetics
*ELAVL1* (ENSG00000066044)	ELAV-like protein 1	bind elements rich in AU in messenger RNA (mRNA); stabilize transcripts	mRNA stability
*ERB4* (ENSG00000178568)	ErbB-4 receptor tyrosine-protein kinase	activate receptor tyrosine kinase signalling; regulate central nervous system development	neuronal development
*FASLG* (ENSG00000117560)	Tumor necrosis factor ligand superfamily member 6, membrane form	bind to the FAS receptor (FAS); induce apoptosis	immune apoptosis
*GABRA1* (ENSG00000067842)	Gamma-aminobutyric acid receptor subunit alpha-1	mediate gamma-aminobutyric acid (GABA) signalling; inhibit neuronal activity	synaptic inhibition
*GALC* (ENSG00000054967)	Galactocerebrosidase	hydrolyze galactosyl ceramide; regulate myelin lipid turnover	myelin metabolism
*GPX6* (ENSG00000143384)	Glutathione peroxidase 6	detoxify peroxides via glutathione peroxidase activity	oxidative stress defence
*GRIA3* (ENSG00000125675)	Glutamate receptor 3	mediate glutamate signalling; enable excitatory neurotransmission	excitotoxicity
*ICOS* (ENSG00000163600)	Inducible T-cell costimulator	enhance T-cell activation; promote antibody production	T-cell activation
*IFI44L* (ENSG00000137965)	Interferon-induced protein 44-like	induce antiviral interferon response	interferon response
*IFNLR1* (ENSG00000185436)	Interferon lambda receptor 1	activate Janus kinase signal transducer and transcription activator (JAK-STAT) signalling	interferon response
*IKZF3* (ENSG00000185811)	Zinc finger protein Aiolos	control lymphocyte differentiation; regulate adaptive immunity	B-cell regulation
*IL10* (ENSG00000136634)	Interleukin-10	suppress inflammatory cytokine production; regulate immune tolerance	immune suppression
*IL6* (ENSG00000136244)	Interleukin-6	induce inflammatory signalling; promote acute phase response	Th17, inflammation
*IRAK1* (ENSG00000184216)	Interleukin-1 receptor-associated kinase 1	mediate Toll-like receptor (TLR) and interleukin-1 receptor (IL1R) signalling	innate immunity
*ITGB8* (ENSG00000105855)	Integrin beta-8	bind fibronectin; recognize the arginine-glycine-aspartate (RGD) motif; activate transforming growth factor beta 1 (TGFB1); regulate vasculogenesis	TGF-β activation, BBB
*KZF2* (ENSG00000185842)	Helios zinc finger protein	regulate T-cell differentiation; maintain regulatory T-cell function	Treg function
*LINGO1* (ENSG00000179886)	Replica-rich repeat and immunoglobulin-like domain-containing nogo receptor-interacting protein 1	inhibit oligodendrocyte differentiation; suppress myelination	remyelination block
*NOS1* (ENSG00000164867)	Nitric oxide synthase, brain	produce nitric oxide (NO); modulate neurotransmission; oxidative stress	neurotoxicity
*NOX4* (ENSG00000138821)	NADPH oxidase 4	generate reactive oxygen species (ROS); modulate intracellular signalling	ROS, neurodegeneration
*OLR1* (ENSG00000173391)	Oxidized low-density lipoprotein receptor 1, soluble form	bind oxidized low-density lipoprotein (oxLDL); activate nuclear factor kappa B (NF-κB)	endothelial dysfunction
*OSM* (ENSG00000164329)	Oncostatin-M	enhance cytokine production: interleukin 6 (IL6); granulocyte colony-stimulating factor (CSF3); granulocyte–macrophage colony-stimulating factor (CSF2); regulate cell proliferation	inflammation, astrocyte activation
*OSMR* (ENSG00000145623)	Oncostatin-M-specific receptor subunit beta	activate signal transducer and activator of transcription (STAT) pathway	inflammation signalling
*PDGFRA* (ENSG00000134853)	Platelet-derived growth factor receptor alpha	activate receptor tyrosine kinase signalling; regulate proliferation; survival; chemotaxis	CNS repair, proliferation
*PDGFRB* (ENSG00000100311)	Platelet-derived growth factor subunit B	stimulate cell proliferation; regulate angiogenesis; vessel maturation	pericytes, BBB
*PLCG2* (ENSG00000197943)	1-Phosphatidylinositol 4,5-bisphosphate phosphodiesterase gamma-2	generate diacylglycerol (DAG) and inositol triphosphate (IP3); propagate signalling	immune signalling
*PRDM1* (ENSG00000140987)	PR domain zinc finger protein 1	repress transcription in lymphocytes; regulate immune cell fate	T/B cell differentiation
*PSMC6* (ENSG00000111671)	Proteasome ATPase	mediate ATP-dependent proteasomal degradation	proteostasis
*PSMD3* (ENSG00000172845)	non-ATPase regulatory subunit 3 of the 26S proteasome	mediate ubiquitin-dependent protein degradation; maintain proteostasis	proteostasis
*PTGS2* (ENSG00000073756)	Prostaglandin G/H synthase 2 / cyclooxygenase 2	convert arachidonic acid to prostaglandins; mediate inflammation	inflammation
*PXDN* (ENSG00000130508)	Peroxidasin homolog	mediate hydrogen peroxide metabolism; support extracellular matrix organisation	oxidative stress, ECM
*RAC1* (ENSG00000136238)	Ras-related C3 botulinum toxin substrate 1	regulate actin cytoskeleton; control cell migration; adhesion	cell migration
*RASGRP1* (ENSG00000172575)	RAS guanyl-releasing protein 1	activate Ras–mitogen-activated protein kinase (MAPK) signalling through guanine nucleotide exchange	T-cell activation
*RORC* (ENSG00000143365)	Nuclear receptor ROR-gamma	drive differentiation of T helper 17 (Th17); regulate immune transcription	Th17 axis
*SH2B3* (ENSG00000169379)	SH2B adapter protein 3	link T-cell receptor (TCR) signalling to downstream pathways	immune regulation
*SOCS1* (ENSG00000185338)	Cytokine signalling suppressor 1	inhibit Janus kinase signal transducer and transcription activator (JAK–STAT) signalling	JAK/STAT inhibition
*SYT1* (ENSG00000123485)	Synaptotagmin-1	sense calcium ions (Ca2+); trigger synaptic vesicle exocytosis	synaptic transmission
*THBS1* (ENSG00000137801)	Thrombospondin-1	mediate cell–cell and cell–matrix adhesion; regulate extracellular interactions	synaptogenesis, BBB
*TNFRSF1B* (ENSG00000028137)	Tumor necrosis factor receptor superfamily member 1b, membrane form	mediate tumor necrosis factor alpha (TNF) signalling	inflammation
*TNFSF10* (ENSG00000121858)	Tumor necrosis factor ligand superfamily member 10	bind tumor necrosis factor receptors (TNFRSF); induce apoptosis	immune-mediated apoptosis
*TRAF6* (ENSG00000175104)	TNF receptor-associated factor 6	activate nuclear factor kappa B (NF-κB) by ubiquitination	inflammation
*USP3* (ENSG00000111850)	Ubiquitin carboxyl-terminal hydrolase 3	remove ubiquitin from histones; regulate DNA repair	DNA repair
*YAP1* (ENSG00000137693)	Transcriptional coactivator YAP1	coactivate transcription; regulate cell proliferation; apoptosis	regeneration

**Table 7:** Characteristics of the miRNA assays used in the study.

Assay Name	MIMAT No.	Mature miRNA Sequence	Location	Assay ID
let-miR-7a-5p	MIMAT0000062	UGAGGUAGUAGGUUGUAUAGUU	Chr. 9	478575_mir
let-miR-7i-5p	MIMAT0000415	UGAGGUAGUAGUUUGUGCUGUU	Chr. 12	478375_mir
hsa-miR-15b-5p	MIMAT0000417	UAGCAGCACAUCAUGGUUUACA	Chr. 3	478313_mir
hsa-miR-16-5p	MIMAT0000069	UAGCAGCACGUAAAUAUUGGCG	Chr. 13	477860_mir
has-miR-21-5p	MIMAT0000076	UAGCUUAUCAGACUGAUGUUGA	Chr. 17	477975_mir
hsa-miR-107	MIMAT0000104	AGCAGCAUUGUACAGGGCUAUCA	Chr. 10	478254_mir
has-miR-126-3p	MIMAT0000445	UCGUACCGUGAGUAAUAAUGCG	Chr. 9	477887_mir
has-miR-142-3p	MIMAT0000434	UGUAGUGUUUCCUACUUUAUGGA	Chr. 9	477910_mir
has-miR-146a-5p	MIMAT0000449	UGAGAACUGAAUUCCAUGGGUU	Chr. 5	478399_mir
has-miR-223-3p	MIMAT0000280	UGAGAACUGAAUUCCAUGGGUU	Chr. X	477983_mir
has-miR-191-5p	MIMAT0000440	CAACGGAAUCCCAAAAGCAGCUG	Chr. 3	477952_mir
cel-miR-39-3p	MIMAT0000010	UCACCGGGUGUAAAUCAGCUUG	-	478293_mir

**Table 9:** Clinical characteristics of the independent external ROC validation cohort characterized by heterogeneous platelet counts in the MS group.

Parameters	Control Group ^a^ (*n* = 22)	MS ^a^ (*n* = 22)
Age [years]	47.5 ± 11.5	51.4 ± 14.3
Gender [F; M]	F: 17 (77%) M: 5 (23%)	F: 12 (59%) M: 9 (41%)
BMI [kg/m^2^]	21.8 ± 1.5	25.8 ± 2.7
Number of blood platelets [× 10^3^/μL]	242.8 ± 78.3 (range: 155–454)	399.9 ± 155.9 (range: 121–623)
MDD [years]	N/A	22.9 ± 7.0
EDSS	N/A	6 (5–6)
mRS	N/A	3 (3–3)
BDI	N/A	14 (11.3–16.8)
MMSE	N/A	25 (23.3–29)
MoCA	N/A	28 (26–29)
ADL	N/A	12 (10.3–14.8)

Legend: ^a^ descriptive statistic: mean ± SD OR median (Q1–Q3) OR number (frequency, %); N/A—not applicable.

## Data Availability

The data generated in this study have been deposited in the University of Lodz Repository and are publicly available under Creative Commons 4.0 at the following link: http://hdl.handle.net/11089/59570.

## Supplementary Materials

The following supporting information can be downloaded at https://www.mdpi.com/article/10.3390/ijms27219914/s1.

## Abbreviations

The following abbreviations are used in this manuscript:

ADL	Activity of daily living
AKT	Protein kinase B
ATP	Adenosine triphosphate
AUC	Area under the curve
BBB	Blood–brain barrier
BDI	Beck Depression Inventory
BDNF	Brain-derived neurotrophic factor
B-H	Benjamini–Hochberg
BMI	Body mass index
CCL19	C-C motif chemokine ligand 19
CI	Confidence interval
CNS	Central nervous system
CPDA-1	Citrate phosphate dextrose adenine-1
CSF2	Granulocyte–macrophage colony-stimulating factor
CSF3	Granulocyte colony-stimulating factor
DAG	Diacylglycerol
DMTs	Disease-modifying therapies
DNA	Deoxyribonucleic acid
ECM	Extracellular matrix
EDSS	Expanded Disability Status Scale
EDTA	Ethylenediaminetetraacetic acid
ELISA	Enzyme-linked immunosorbent assay
ErbB	Erythroblastic leukemia viral oncogene homolog
EVs	Extracellular vesicles
FC	Fold change
FGF-2	Fibroblast growth factor 2
FMO	Fluorescence-minus-one
FSC	Forward scatter
GABA	Gamma-aminobutyric acid
GO	Gene ontology
GPVI	Glycoprotein VI
H_2_O_2_	Hydrogen peroxide
H3K9	Histone H3 lysine 9
IGF1R	Insulin-like growth factor 1 receptor
IL	Interleukin
IL1R	Interleukin-1 receptor
IL6	Interleukin-6
iNOS	Inducible nitric oxide synthase
IP3	Inositol 1,4,5-trisphosphate
JAK	Janus kinase
JAK–STAT	Janus kinase–signal transducer and activator of transcription
KEGG	Kyoto Encyclopedia of Genes and Genomes
MAPK	Mitogen-activated protein kinase
MDD	Mean disease duration
miRNA	Micro RNA
MMSE	Mini-Mental State Examination
MoCA	Montreal Cognitive Assessment
mRNA	Messenger RNA
MRI	Magnetic resonance imaging
mRS	Modified Rankin Scale
MS	Multiple sclerosis
Mt	Mitochondria
NF-κB	Nuclear factor kappa B
NO	Nitric oxide
NOS	Nitric oxide synthase
NSAID	Nonsteroidal anti-inflammatory drugs
OCS	Open canalicular system
oxLDL	Oxidized low-density lipoprotein
P	Pseudopodia
PAGs	Platelet aggregates
PBMCs	Peripheral blood mononuclear cells
PDGF	Platelet-derived growth factor
PGE1	Prostaglandin E1
PI3K	Phosphoinositide 3-kinase
PI3K/AKT	Phosphoinositide 3-kinase/protein kinase B
PMPs	Platelet microparticles
PPI	Protein–protein interaction
PRP	Platelet-rich plasma
PPMS	Primary progressive multiple sclerosis
Q	Quartile
RGD	Arginine-glycine-aspartate
RISC	RNA-induced silencing complex
RNA	Ribonucleic acid
ROC	Receiver operating characteristic
ROS	Reactive oxygen species
RRMS	Relapsing–remitting multiple sclerosis
RT	Room temperature
RT-PCR	Reverse transcription polymerase chain reaction
SD	Standard deviation
Ser/Thr	Serine/threonine
SIRT1	Sirtuin 1
SPMS	Secondary progressive multiple sclerosis
SSRIs	Selective serotonin reuptake inhibitors
STAT	Signal transducer and activator of transcription
TCR	T-cell receptor
TEM	Transmission electron microscopy
TGF-β	Transforming growth factor beta
TGFB1	Transforming growth factor beta 1
Th17	T helper 17
TLR	Toll-like receptor
TNF	Tumor necrosis factor
TNFRSF	Tumor necrosis factor receptor superfamily
VEGFA	Vascular endothelial growth factor A
VSMCs	Vascular smooth muscle cells
