# Supplementary material for: Platelet miRNA-Based Signature as a Potential Non-Invasive Tool for Discriminating Patients with Secondary Progressive Multiple Sclerosis from Healthy Individuals
Source: Int J Mol Sci. 2026 Nov 5;27(21):9914. doi: 10.3390/ijms27219914 (PMC13763952; doi:10.3390/ijms27219914)
Supplement: Supplementary file 1 [file ijms-27-09914-s001.zip › Figure S2.pdf]

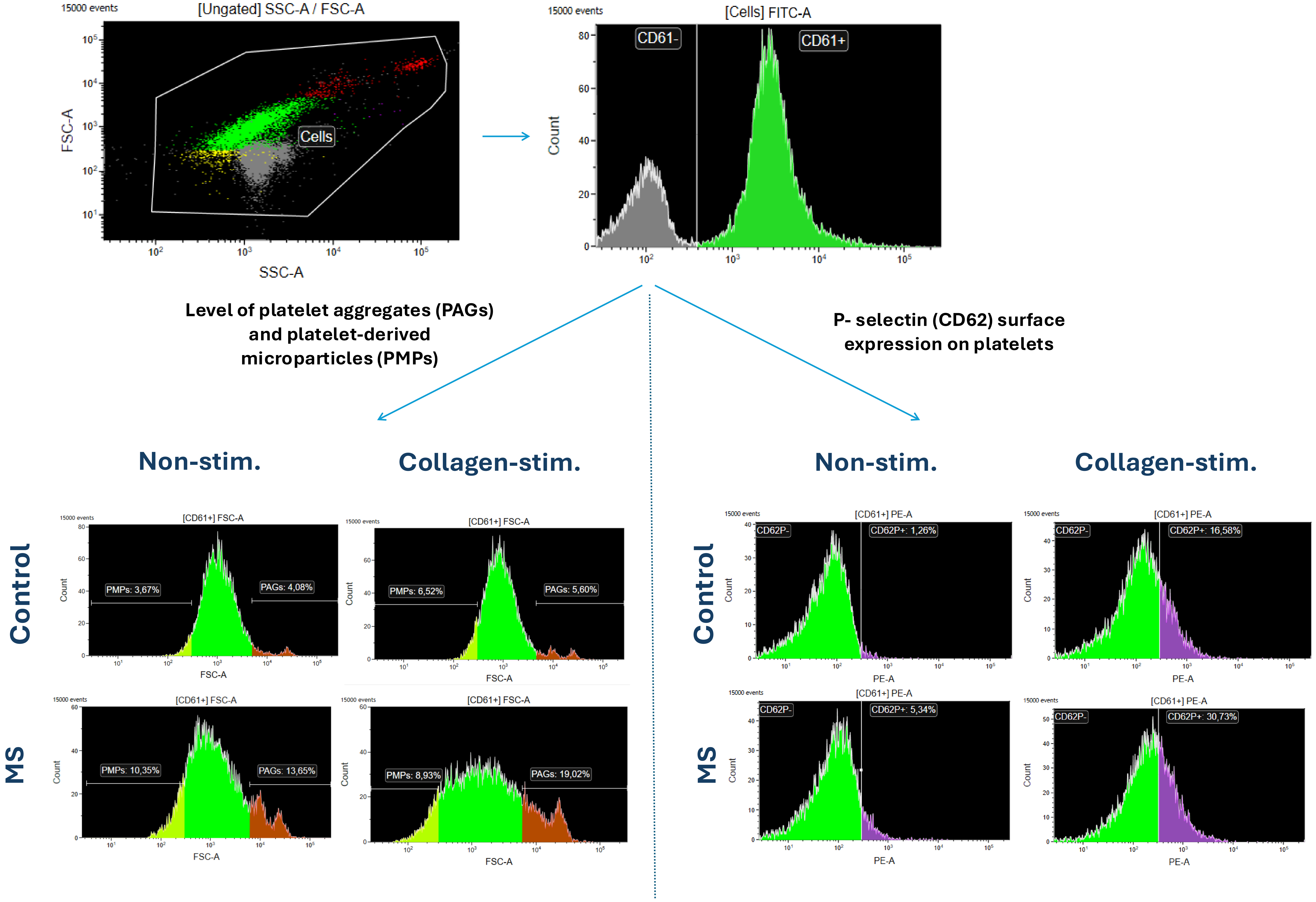
15000 events
[Ungated] SSC-A / FSC-A
FSC-A
SSC-A
Cells
15000 events
[Cells] FITC-A
CD61-
CD61+
Count
Level of platelet aggregates (PAGs) and platelet-derived microparticles (PMPs)
P- selectin (CD62) surface expression on platelets
Non-stim.
Collagen-stim.
Non-stim.
Collagen-stim.
Control
MS
Control
MS
[CD61+] FSC-A
PMPs: 3,67%
PAGs: 4,08%
PMPs: 6,52%
PAGs: 5,60%
PMPs: 10,35%
PAGs: 13,65%
PMPs: 8,93%
PAGs: 19,02%
FSC-A
[CD61+] PE-A
CD62P-
CD62P+: 1,26%
CD62P+: 16,58%
CD62P+: 5,34%
CD62P+: 30,73%
PE-A
