# Supplementary material for: Platelet miRNA-Based Signature as a Potential Non-Invasive Tool for Discriminating Patients with Secondary Progressive Multiple Sclerosis from Healthy Individuals
Source: Int J Mol Sci. 2026 Nov 5;27(21):9914. doi: 10.3390/ijms27219914 (PMC13763952; doi:10.3390/ijms27219914)
Supplement: Supplementary file 1 [file ijms-27-09914-s001.zip › Table S1.pdf]

BLOOD PLATELETS

Systematic_name	p ([MS] Vs [Control])	p (Corr) ([MS] Vs [Control])	Log FC ([MS] Vs [Control])	FC (abs) ([MS] Vs [Control])	Regulation ([MS] Vs [Control])	FC ([MS] Vs [Control])
**hsa-let-7a-5p**	**0.0011**	**0.0033**	**-4.32**	**19.98**	**down**	**-19.98**
hsa-let-7b-3p	0.0015	0.0034	3.78	13.74	up	13.74
hsa-let-7d-5p	0.0007	0.0026	-6.25	76.01	down	-76.01
hsa-let-7e-5p	0.0013	0.0034	-4.42	21.44	down	-21.44
hsa-let-7f-1-3p	0.0020	0.0041	4.05	16.56	up	16.56
hsa-let-7f-5p	0.0003	0.0022	-7.56	188.97	down	-188.97
hsa-let-7g-5p	0.0002	0.0021	-6.71	104.94	down	-104.94
**hsa-let-7i-5p**	**0.0066**	**0.0084**	**-1.43**	**2.69**	**down**	**-2.69**
hsa-miR-103a-3p	0.0008	0.0028	-5.28	38.80	down	-38.80
hsa-miR-106b-5p	0.0034	0.0052	-2.47	5.54	down	-5.54
**hsa-miR-107**	**0.0006**	**0.0025**	**-4.57**	**23.77**	**down**	**-23.77**
hsa-miR-1202	0.0132	0.0147	-1.75	3.36	down	-3.36
hsa-miR-1228-3p	0.0013	0.0034	3.62	12.31	up	12.31
hsa-miR-1234-3p	0.0020	0.0041	3.62	12.33	up	12.33
hsa-miR-1237-3p	0.0226	0.0239	4.06	16.66	up	16.66
hsa-miR-1238-3p	0.0043	0.0062	3.75	13.49	up	13.49
hsa-miR-124-3p	0.0035	0.0053	7.73	211.79	up	211.79
hsa-miR-125b-1-3p	0.0207	0.0220	-1.33	2.51	down	-2.51
**hsa-miR-126-3p**	**0.0009**	**0.0028**	**-5.13**	**35.06**	**down**	**-35.06**
hsa-miR-1273g-3p	0.0024	0.0043	2.19	4.55	up	4.55
hsa-miR-1281	0.0021	0.0041	3.35	10.17	up	10.17
hsa-miR-129-2-3p	0.0023	0.0042	4.13	17.53	up	17.53
hsa-miR-1304-3p	0.0011	0.0033	3.76	13.54	up	13.54
hsa-miR-130a-3p	0.0006	0.0025	-3.96	15.56	down	-15.56
hsa-miR-140-5p	0.0108	0.0124	-1.40	2.63	down	-2.63
**hsa-miR-142-3p**	**0.0014**	**0.0034**	**-1.96**	**3.88**	**down**	**-3.88**
**hsa-miR-146a-5p**	**0.0023**	**0.0042**	**-2.51**	**5.71**	**down**	**-5.71**

hsa-miR-1470	0.0001	0.0021	8.58	382.43	up	382.43
hsa-miR-149-5p	0.0006	0.0025	3.94	15.33	up	15.33
hsa-miR-150-5p	0.0118	0.0133	-2.07	4.19	down	-4.19
hsa-miR-151a-5p	0.0002	0.0021	-3.74	13.40	down	-13.40
hsa-miR-151b	0.0005	0.0025	-1.73	3.32	down	-3.32
hsa-miR-1539	0.0021	0.0041	4.05	16.53	up	16.53
hsa-miR-1587	0.0060	0.0080	4.03	16.28	up	16.28
hsa-miR-15a-5p	0.0002	0.0021	-4.58	23.97	down	-23.97
**hsa-miR-15b-5p**	**0.0003**	**0.0022**	**-5.31**	**39.65**	**down**	**-39.65**
**hsa-miR-16-5p**	**0.0005**	**0.0025**	**-5.88**	**59.06**	**down**	**-59.06**
hsa-miR-17-5p	0.0016	0.0035	-3.84	14.35	down	-14.35
hsa-miR-181a-5p	0.0079	0.0094	1.06	2.09	up	2.09
hsa-miR-1825	0.0024	0.0043	3.39	10.48	up	10.48
hsa-miR-185-5p	0.0059	0.0080	-1.60	3.04	down	-3.04
hsa-miR-1914-3p	0.0002	0.0021	10.94	1967.45	up	1967.45
hsa-miR-197-3p	0.0084	0.0098	2.13	4.37	up	4.37
hsa-miR-197-5p	0.0047	0.0067	4.08	16.87	up	16.87
hsa-miR-199a-3p	0.0004	0.0022	-5.30	39.33	down	-39.33
hsa-miR-199a-5p	0.0014	0.0034	-1.54	2.90	down	-2.90
hsa-miR-19b-3p	0.0031	0.0049	-1.99	3.96	down	-3.96
hsa-miR-20a-5p	0.0004	0.0022	-5.13	35.00	down	-35.00
hsa-miR-20b-5p	0.0052	0.0073	-2.22	4.64	down	-4.64
hsa-miR-2116-3p	0.0026	0.0045	4.04	16.41	up	16.41
**hsa-miR-21-3p**	**0.0000**	**0.0005**	**-3.37**	**10.36**	**down**	**-10.36**
**hsa-miR-223-3p**	**0.0006**	**0.0025**	**-4.39**	**21.03**	**down**	**-21.03**
hsa-miR-23a-3p	0.0004	0.0022	-3.87	14.60	down	-14.60
hsa-miR-23c	0.0018	0.0038	4.04	16.42	up	16.42
hsa-miR-24-3p	0.0007	0.0026	-5.01	32.33	down	-32.33
hsa-miR-25-3p	0.0023	0.0042	-3.07	8.38	down	-8.38
hsa-miR-26a-5p	0.0008	0.0027	-4.29	19.55	down	-19.55
hsa-miR-26b-5p	0.0005	0.0023	-3.49	11.22	down	-11.22
hsa-miR-27a-3p	0.0003	0.0022	-3.91	15.07	down	-15.07
hsa-miR-27b-3p	0.0004	0.0022	-2.90	7.48	down	-7.48
hsa-miR-30d-5p	0.0007	0.0026	4.45	21.89	up	21.89

hsa-miR-3135b	0.0015	0.0034	5.79	55.42	up	55.42
hsa-miR-3150b-5p	0.0057	0.0078	7.97	250.53	up	250.53
hsa-miR-3151-3p	0.0025	0.0043	2.25	4.74	up	4.74
hsa-miR-3162-3p	0.0022	0.0042	2.86	7.28	up	7.28
hsa-miR-3162-5p	0.0014	0.0034	3.02	8.12	up	8.12
hsa-miR-3180-5p	0.0116	0.0132	4.23	18.82	up	18.82
hsa-miR-3184-3p	0.0067	0.0084	7.88	235.56	up	235.56
hsa-miR-3190-5p	0.0062	0.0081	8.19	292.84	up	292.84
hsa-miR-320a	0.0013	0.0034	-4.22	18.65	down	-18.65
hsa-miR-324-3p	0.0018	0.0038	-1.90	3.73	down	-3.73
hsa-miR-324-5p	0.0027	0.0046	-2.00	4.00	down	-4.00
hsa-miR-328-3p	0.0001	0.0021	-1.29	2.44	down	-2.44
hsa-miR-331-3p	0.0002	0.0021	-4.74	26.72	down	-26.72
hsa-miR-33b-3p	0.0043	0.0062	4.23	18.77	up	18.77
hsa-miR-342-5p	0.0011	0.0033	-1.34	2.53	down	-2.53
hsa-miR-3591-3p	0.0032	0.0051	-1.67	3.17	down	-3.17
hsa-miR-3613-3p	0.0006	0.0025	3.73	13.29	up	13.29
hsa-miR-3656	0.0038	0.0056	4.09	16.99	up	16.99
hsa-miR-365a-3p	0.0003	0.0022	4.99	31.79	up	31.79
hsa-miR-3960	0.0029	0.0048	4.14	17.68	up	17.68
hsa-miR-423-5p	0.0014	0.0034	-3.16	8.91	down	-8.91
hsa-miR-425-3p	0.0023	0.0042	3.84	14.30	up	14.30
hsa-miR-4254	0.0035	0.0053	-1.30	2.46	down	-2.46
hsa-miR-4284	0.0035	0.0053	2.26	4.78	up	4.78
hsa-miR-4306	0.0057	0.0078	-1.78	3.43	down	-3.43
hsa-miR-4310	0.0044	0.0063	3.92	15.17	up	15.17
hsa-miR-4313	0.0017	0.0037	3.61	12.22	up	12.22
hsa-miR-4323	0.0002	0.0021	3.47	11.10	up	11.10
hsa-miR-432-5p	0.0019	0.0040	-4.41	21.32	down	-21.32
hsa-miR-4433a-5p	0.0023	0.0042	3.26	9.56	up	9.56
hsa-miR-4436b-5p	0.0059	0.0080	3.78	13.77	up	13.77
hsa-miR-4455	0.0004	0.0022	-2.18	4.52	down	-4.52
hsa-miR-4459	0.0018	0.0038	5.62	49.15	up	49.15
hsa-miR-4478	0.0013	0.0034	7.99	254.64	up	254.64

hsa-miR-4485-3p	0.0008	0.0027	5.07	33.54	up	33.54
hsa-miR-4485-5p	0.0062	0.0081	2.07	4.20	up	4.20
hsa-miR-4505	0.0061	0.0080	3.69	12.88	up	12.88
hsa-miR-4507	0.0096	0.0111	2.84	7.16	up	7.16
hsa-miR-4515	0.0005	0.0025	4.13	17.45	up	17.45
hsa-miR-451a	0.0007	0.0026	-2.45	5.45	down	-5.45
hsa-miR-451b	0.0002	0.0021	-2.95	7.75	down	-7.75
hsa-miR-4530	0.0107	0.0124	3.92	15.13	up	15.13
hsa-miR-4636	0.0015	0.0035	7.98	252.94	up	252.94
hsa-miR-4649-3p	0.0037	0.0055	3.35	10.22	up	10.22
hsa-miR-4652-3p	0.0004	0.0022	3.72	13.14	up	13.14
hsa-miR-4664-3p	0.0245	0.0256	4.18	18.07	up	18.07
hsa-miR-4665-3p	0.0025	0.0043	3.72	13.20	up	13.20
hsa-miR-4666b	0.0012	0.0033	4.06	16.72	up	16.72
hsa-miR-4687-3p	0.0036	0.0053	3.69	12.95	up	12.95
hsa-miR-4697-5p	0.0007	0.0026	8.40	338.33	up	338.33
hsa-miR-4698	0.0005	0.0025	8.52	366.27	up	366.27
hsa-miR-4701-5p	0.0004	0.0022	3.10	8.60	up	8.60
hsa-miR-4721	0.0008	0.0028	9.56	752.91	up	752.91
hsa-miR-4725-5p	0.0055	0.0077	3.66	12.65	up	12.65
hsa-miR-4728-3p	0.0256	0.0267	3.89	14.80	up	14.80
hsa-miR-4730	0.0000	0.0011	-3.93	15.28	down	-15.28
hsa-miR-4731-3p	0.0004	0.0022	3.77	13.63	up	13.63
hsa-miR-4749-3p	0.0028	0.0047	3.80	13.96	up	13.96
hsa-miR-4758-3p	0.0022	0.0042	3.74	13.40	up	13.40
hsa-miR-4769-3p	0.0020	0.0040	3.34	10.15	up	10.15
hsa-miR-4787-3p	0.0012	0.0033	3.76	13.53	up	13.53
hsa-miR-4787-5p	0.0025	0.0043	3.64	12.49	up	12.49
hsa-miR-483-3p	0.0030	0.0049	-1.29	2.44	down	-2.44
hsa-miR-494-3p	0.0077	0.0092	2.44	5.43	up	5.43
hsa-miR-550a-5p	0.0081	0.0096	4.10	17.10	up	17.10
hsa-miR-563	0.0155	0.0170	4.51	22.84	up	22.84
hsa-miR-574-3p	0.0003	0.0022	-3.52	11.51	down	-11.51
hsa-miR-6069	0.0034	0.0052	3.53	11.57	up	11.57

hsa-miR-6073	0.0000	0.0011	-3.82	14.08	down	-14.08
hsa-miR-6087	0.0110	0.0126	2.62	6.14	up	6.14
hsa-miR-6088	0.0026	0.0045	5.62	49.30	up	49.30
hsa-miR-6089	0.0035	0.0053	3.54	11.59	up	11.59
hsa-miR-6125	0.0180	0.0194	2.18	4.55	up	4.55
hsa-miR-6127	0.0033	0.0051	2.91	7.51	up	7.51
hsa-miR-6165	0.0071	0.0086	-2.02	4.06	down	-4.06
hsa-miR-634	0.0011	0.0033	3.91	15.07	up	15.07
hsa-miR-638	0.0378	0.0378	-1.13	2.19	down	-2.19
hsa-miR-642a-3p	0.0055	0.0077	3.33	10.03	up	10.03
hsa-miR-6507-3p	0.0007	0.0026	4.16	17.86	up	17.86
hsa-miR-6508-5p	0.0028	0.0046	3.86	14.48	up	14.48
hsa-miR-6514-3p	0.0000	0.0005	-1.69	3.23	down	-3.23
hsa-miR-6515-3p	0.0002	0.0021	3.63	12.39	up	12.39
hsa-miR-652-3p	0.0002	0.0021	-1.51	2.84	down	-2.84
hsa-miR-664b-5p	0.0001	0.0021	7.94	245.81	up	245.81
hsa-miR-6716-3p	0.0003	0.0022	-4.04	16.48	down	-16.48
hsa-miR-6731-3p	0.0374	0.0375	3.44	10.86	up	10.86
hsa-miR-6737-3p	0.0002	0.0021	3.79	13.82	up	13.82
hsa-miR-6739-5p	0.0012	0.0034	-1.47	2.78	down	-2.78
hsa-miR-6752-3p	0.0019	0.0040	4.71	26.16	up	26.16
hsa-miR-6756-3p	0.0002	0.0021	3.71	13.08	up	13.08
hsa-miR-6757-3p	0.0285	0.0290	4.51	22.73	up	22.73
hsa-miR-6759-3p	0.0065	0.0083	-1.33	2.52	down	-2.52
hsa-miR-6760-3p	0.0012	0.0033	3.63	12.37	up	12.37
hsa-miR-6763-3p	0.0012	0.0034	3.63	12.38	up	12.38
hsa-miR-6766-3p	0.0008	0.0027	3.61	12.17	up	12.17
hsa-miR-6777-3p	0.0228	0.0240	4.22	18.64	up	18.64
hsa-miR-6785-3p	0.0162	0.0176	3.15	8.91	up	8.91
hsa-miR-6785-5p	0.0027	0.0045	6.30	78.74	up	78.74
hsa-miR-6795-3p	0.0063	0.0082	3.68	12.84	up	12.84
hsa-miR-6797-3p	0.0030	0.0048	3.58	11.92	up	11.92
hsa-miR-6798-3p	0.0017	0.0037	3.70	13.02	up	13.02
hsa-miR-6800-3p	0.0013	0.0034	3.83	14.22	up	14.22

hsa-miR-6800-5p	0.0055	0.0077	4.32	20.01	up	20.01
hsa-miR-6803-5p	0.0085	0.0100	2.59	6.00	up	6.00
hsa-miR-6812-3p	0.0070	0.0086	4.04	16.46	up	16.46
hsa-miR-6813-3p	0.0011	0.0033	3.24	9.42	up	9.42
hsa-miR-6819-3p	0.0066	0.0084	4.12	17.36	up	17.36
hsa-miR-6824-3p	0.0014	0.0034	3.62	12.29	up	12.29
hsa-miR-6834-3p	0.0265	0.0275	2.48	5.57	up	5.57
hsa-miR-6848-3p	0.0021	0.0041	3.81	14.06	up	14.06
hsa-miR-6851-3p	0.0004	0.0023	3.69	12.93	up	12.93
hsa-miR-6858-3p	0.0001	0.0021	4.10	17.18	up	17.18
hsa-miR-6865-3p	0.0020	0.0041	3.86	14.49	up	14.49
hsa-miR-6869-5p	0.0014	0.0034	4.79	27.70	up	27.70
hsa-miR-6870-3p	0.0001	0.0021	3.88	14.68	up	14.68
hsa-miR-6877-3p	0.0067	0.0084	7.91	240.01	up	240.01
hsa-miR-6880-3p	0.0017	0.0037	3.50	11.32	up	11.32
hsa-miR-6889-3p	0.0037	0.0054	3.87	14.59	up	14.59
hsa-miR-7107-5p	0.0023	0.0042	3.54	11.63	up	11.63
hsa-miR-744-5p	0.0013	0.0034	-3.00	8.00	down	-8.00
hsa-miR-7641	0.0000	0.0011	6.58	95.86	up	95.86
hsa-miR-766-3p	0.0061	0.0080	2.98	7.86	up	7.86
hsa-miR-767-3p	0.0029	0.0048	7.93	243.28	up	243.28
hsa-miR-7704	0.0149	0.0164	2.60	6.06	up	6.06
hsa-miR-7974	0.0137	0.0152	4.34	20.28	up	20.28
hsa-miR-7977	0.0071	0.0086	-1.79	3.46	down	-3.46
hsa-miR-8069	0.0296	0.0300	1.17	2.25	up	2.25
hsa-miR-8089	0.0002	0.0021	8.57	380.69	up	380.69
hsa-miR-8485	0.0002	0.0021	-3.30	9.88	down	-9.88
hsa-miR-874-3p	0.0014	0.0034	9.80	892.47	up	892.47
hsa-miR-933	0.0004	0.0022	3.55	11.68	up	11.68
hsa-miR-93-5p	0.0012	0.0034	-3.90	14.92	down	-14.92
hsa-miR-940	0.0020	0.0041	3.53	11.58	up	11.58
hsa-miR-98-3p	0.0003	0.0022	4.14	17.59	up	17.59
hsa-miR-98-5p	0.0009	0.0029	-1.67	3.19	down	-3.19

MEGAKARYOCYTES

Systematic_name	p ([PMS] Vs [Control])	p (Corr) ([PMS] Vs [Control])	Log FC ([PMS] Vs [Control])	FC (abs) ([PMS] Vs [Control])	Regulation ([PMS] Vs [Control])	FC ([PMS] Vs [Control])
**hsa-let-7a-5p**	**0.0014**	**0.0022**	**-1.17**	**2.24**	**down**	**-2.24**
hsa-let-7b-3p	0.0007	0.0013	-1.45	2.73	down	-2.73
hsa-let-7e-5p	0.0000	0.0000	2.36	5.14	up	5.14
hsa-let-7f-1-3p	0.0093	0.0107	-1.08	2.12	down	-2.12
**hsa-let-7i-5p**	**0.0029**	**0.0040**	**-1.07**	**2.10**	**down**	**-2.10**
hsa-miR-103a-3p	0.0000	0.0000	5.38	41.65	up	41.65
hsa-miR-106b-5p	0.0000	0.0000	3.59	12.02	up	12.02
**hsa-miR-107**	**0.0000**	**0.0000**	**4.25**	**19.02**	**up**	**19.02**
hsa-miR-1183	0.0000	0.0000	2.75	6.73	up	6.73
hsa-miR-1202	0.0011	0.0019	-1.19	2.28	down	-2.28
hsa-miR-1224-5p	0.0000	0.0000	3.02	8.09	up	8.09
hsa-miR-1228-3p	0.0016	0.0024	-1.19	2.29	down	-2.29
hsa-miR-1234-3p	0.0005	0.0011	-1.43	2.70	down	-2.70
hsa-miR-1236-5p	0.0020	0.0029	2.88	7.36	up	7.36
hsa-miR-1238-3p	0.0028	0.0039	-1.27	2.42	down	-2.42
hsa-miR-1249-3p	0.0019	0.0029	-1.12	2.18	down	-2.18
hsa-miR-1260a	0.0256	0.0264	2.03	4.09	up	4.09
hsa-miR-1260b	0.0000	0.0000	3.09	8.49	up	8.49
**hsa-miR-126-3p**	**0.0000**	**0.0000**	**5.61**	**48.81**	**up**	**48.81**
hsa-miR-1273g-3p	0.0013	0.0021	1.22	2.33	up	2.33
hsa-miR-1275	0.0021	0.0030	1.17	2.25	up	2.25
hsa-miR-1281	0.0022	0.0031	-1.15	2.21	down	-2.21
hsa-miR-129-2-3p	0.0014	0.0022	-1.28	2.43	down	-2.43
hsa-miR-1304-3p	0.0063	0.0077	-1.14	2.20	down	-2.20
hsa-miR-130a-3p	0.0000	0.0000	4.78	27.50	up	27.50
hsa-miR-134-5p	0.0000	0.0000	4.61	24.47	up	24.47
hsa-miR-135a-3p	0.0060	0.0075	2.30	4.92	up	4.92

**hsa-miR-142-3p**	**0.0002**	**0.0004**	**1.72**	**3.30**	**up**	**3.30**
hsa-miR-142-5p	0.0000	0.0000	4.34	20.22	up	20.22
**hsa-miR-146a-5p**	**0.0000**	**0.0000**	**3.26**	**9.57**	**up**	**9.57**
hsa-miR-146b-5p	0.0000	0.0000	3.17	9.02	up	9.02
hsa-miR-149-5p	0.0012	0.0020	-1.38	2.60	down	-2.60
hsa-miR-151a-5p	0.0000	0.0000	3.04	8.20	up	8.20
hsa-miR-1587	0.0025	0.0035	1.15	2.21	up	2.21
hsa-miR-15a-5p	0.0000	0.0000	5.18	36.13	up	36.13
**hsa-miR-15b-5p**	**0.0000**	**0.0001**	**2.12**	**4.36**	**up**	**4.36**
**hsa-miR-16-5p**	**0.0000**	**0.0000**	**2.21**	**4.62**	**up**	**4.62**
hsa-miR-17-5p	0.0000	0.0000	4.08	16.92	up	16.92
hsa-miR-181a-5p	0.0062	0.0076	2.59	6.03	up	6.03
hsa-miR-1825	0.0011	0.0018	-1.28	2.43	down	-2.43
hsa-miR-188-5p	0.0258	0.0264	2.65	6.29	up	6.29
hsa-miR-1972	0.0003	0.0006	-1.47	2.77	down	-2.77
hsa-miR-199a-3p	0.0000	0.0000	4.74	26.65	up	26.65
hsa-miR-199a-5p	0.0000	0.0000	2.85	7.22	up	7.22
hsa-miR-19a-3p	0.0000	0.0000	4.31	19.77	up	19.77
hsa-miR-19b-3p	0.0000	0.0000	5.80	55.90	up	55.90
hsa-miR-20a-5p	0.0000	0.0000	5.29	39.01	up	39.01
hsa-miR-20b-5p	0.0000	0.0000	2.59	6.01	up	6.01
**hsa-miR-21-5p**	**0.0000**	**0.0000**	**4.13**	**17.47**	**up**	**17.47**
hsa-miR-21-5p	0.0000	0.0000	2.55	5.86	up	5.86
**hsa-miR-223-3p**	**0.0000**	**0.0000**	**2.23**	**4.69**	**up**	**4.69**
hsa-miR-22-3p	0.0000	0.0000	4.49	22.43	up	22.43
hsa-miR-23a-3p	0.0001	0.0002	4.56	23.66	up	23.66
hsa-miR-23b-3p	0.0000	0.0000	2.58	5.97	up	5.97
hsa-miR-24-3p	0.0000	0.0000	5.50	45.29	up	45.29
hsa-miR-25-3p	0.0000	0.0000	3.83	14.24	up	14.24
hsa-miR-26a-5p	0.0000	0.0000	5.64	49.79	up	49.79

hsa-miR-26b-5p	0.0000	0.0000	5.24	37.86	up	37.86
hsa-miR-27a-3p	0.0000	0.0000	5.45	43.68	up	43.68
hsa-miR-29a-3p	0.0000	0.0000	5.42	42.88	up	42.88
hsa-miR-29b-3p	0.0000	0.0000	4.29	19.50	up	19.50
hsa-miR-29c-3p	0.0000	0.0000	4.54	23.32	up	23.32
hsa-miR-30b-5p	0.0000	0.0000	4.05	16.58	up	16.58
hsa-miR-30c-5p	0.0000	0.0000	2.68	6.43	up	6.43
hsa-miR-30d-5p	0.0000	0.0000	3.99	15.89	up	15.89
hsa-miR-3138	0.0056	0.0070	2.46	5.49	up	5.49
hsa-miR-3151-3p	0.0008	0.0014	-1.30	2.47	down	-2.47
hsa-miR-3162-3p	0.0013	0.0022	-1.30	2.47	down	-2.47
hsa-miR-3195	0.0000	0.0000	4.49	22.39	up	22.39
hsa-miR-320a	0.0000	0.0000	3.34	10.14	up	10.14
hsa-miR-328-5p	0.0000	0.0000	2.38	5.22	up	5.22
hsa-miR-342-3p	0.0053	0.0067	2.81	7.01	up	7.01
hsa-miR-345-3p	0.0000	0.0000	2.80	6.98	up	6.98
hsa-miR-3613-3p	0.0205	0.0216	-1.07	2.09	down	-2.09
hsa-miR-3648	0.0000	0.0000	3.44	10.88	up	10.88
hsa-miR-3652	0.0015	0.0023	3.26	9.56	up	9.56
hsa-miR-3653-3p	0.0001	0.0002	-1.72	3.30	down	-3.30
hsa-miR-3911	0.0067	0.0081	2.54	5.82	up	5.82
hsa-miR-3940-5p	0.0000	0.0000	2.69	6.47	up	6.47
hsa-miR-423-5p	0.0178	0.0189	1.99	3.96	up	3.96
hsa-miR-424-5p	0.0000	0.0000	2.60	6.08	up	6.08
hsa-miR-425-3p	0.0024	0.0033	-1.26	2.39	down	-2.39
hsa-miR-425-5p	0.0000	0.0000	2.93	7.62	up	7.62
hsa-miR-4274	0.0000	0.0000	2.98	7.90	up	7.90
hsa-miR-4306	0.0000	0.0000	3.35	10.17	up	10.17
hsa-miR-4313	0.0004	0.0008	-1.40	2.63	down	-2.63
hsa-miR-4323	0.0062	0.0076	-1.07	2.10	down	-2.10

hsa-miR-4327	0.0122	0.0135	2.39	5.26	up	5.26
hsa-miR-4433a-3p	0.0000	0.0000	4.38	20.79	up	20.79
hsa-miR-4433a-5p	0.0012	0.0020	-1.33	2.52	down	-2.52
hsa-miR-4436b-3p	0.0000	0.0000	3.24	9.43	up	9.43
hsa-miR-4443	0.0000	0.0000	2.30	4.93	up	4.93
hsa-miR-4463	0.0000	0.0000	4.18	18.10	up	18.10
hsa-miR-4465	0.0002	0.0004	-1.53	2.90	down	-2.90
hsa-miR-4485-5p	0.0006	0.0011	-1.33	2.51	down	-2.51
hsa-miR-4486	0.0001	0.0002	-1.70	3.24	down	-3.24
hsa-miR-451a	0.0000	0.0000	4.54	23.25	up	23.25
hsa-miR-4532	0.0011	0.0019	3.41	10.62	up	10.62
hsa-miR-4534	0.0113	0.0126	2.81	7.00	up	7.00
hsa-miR-4646-5p	0.0011	0.0019	3.25	01,09,4842	up	01,09,4842
hsa-miR-4649-3p	0.0010	0.0018	-1.35	2.54	down	-2.54
hsa-miR-4651	0.0000	0.0000	2.53	5.79	up	5.79
hsa-miR-4665-3p	0.0056	0.0070	-1.03	2.04	down	-2.04
hsa-miR-4665-5p	0.0000	0.0001	3.13	8.78	up	8.78
hsa-miR-4667-5p	0.0072	0.0085	2.64	6.23	up	6.23
hsa-miR-4689	0.0000	0.0001	2.71	6.55	up	6.55
hsa-miR-4697-5p	0.0038	0.0050	-1.03	2.04	down	-2.04
hsa-miR-4698	0.0000	0.0000	3.64	12.46	up	12.46
hsa-miR-4701-3p	0.0225	0.0233	2.09	4.27	up	4.27
hsa-miR-4701-5p	0.0003	0.0008	-1.48	2.79	down	-2.79
hsa-miR-4710	0.0000	0.0000	3.71	13.05	up	13.05
hsa-miR-4716-3p	0.0000	0.0000	2.80	6.95	up	6.95
hsa-miR-4725-5p	0.0040	0.0053	-1.23	2.34	down	-2.34
hsa-miR-4730	0.0000	0.0001	3.00	7.99	up	7.99
hsa-miR-4731-3p	0.0005	0.0010	-1.44	2.72	down	-2.72
hsa-miR-4738-3p	0.0006	0.0013	-1.29	2.44	down	-2.44
hsa-miR-4743-5p	0.0000	0.0000	2.89	7.40	up	7.40

hsa-miR-4749-3p	0.0012	0.0019	-1.25	2.37	down	-2.37
hsa-miR-4750-3p	0.0004	0.0009	-1.42	2.68	down	-2.68
hsa-miR-4767	0.0201	0.0212	2.16	4.47	up	4.47
hsa-miR-4769-3p	0.0005	0.0010	-1.39	2.62	down	-2.62
hsa-miR-4787-3p	0.0049	0.0062	-1.01	2.01	down	-2.01
hsa-miR-483-3p	0.0021	0.0030	-1.34	2.53	down	-2.53
hsa-miR-484	0.0000	0.0001	2.55	5.84	up	5.84
hsa-miR-486-5p	0.0000	0.0000	3.31	9.89	up	9.89
hsa-miR-5010-3p	0.0000	0.0000	2.96	7.80	up	7.80
hsa-miR-5195-3p	0.0000	0.0000	3.54	11.61	up	11.61
hsa-miR-548q	0.0000	0.0001	2.75	6.71	up	6.71
hsa-miR-5703	0.0021	0.0030	3.11	8.62	up	8.62
hsa-miR-574-3p	0.0003	0.0007	-1.42	2.68	down	-2.68
hsa-miR-575	0.0004	0.0009	1.57	2.97	up	2.97
hsa-miR-6069	0.0042	0.0056	-1.14	2.20	down	-2.20
hsa-miR-6075	0.0000	0.0000	2.68	6.39	up	6.39
hsa-miR-6086	0.0020	0.0030	3.06	8.33	up	8.33
hsa-miR-6126	0.0001	0.0001	-1.75	3.36	down	-3.36
hsa-miR-619-5p	0.0023	0.0032	-1.08	2.11	down	-2.11
hsa-miR-630	0.0082	0.0096	3.48	11.17	up	11.17
hsa-miR-634	0.0095	0.0108	-1.10	2.15	down	-2.15
hsa-miR-6507-3p	0.0101	0.0113	-1.11	2.15	down	-2.15
hsa-miR-6508-5p	0.0041	0.0055	-1.25	2.39	down	-2.39
hsa-miR-6515-3p	0.0014	0.0022	-1.27	2.41	down	-2.41
hsa-miR-652-5p	0.0000	0.0000	3.01	8.03	up	8.03
hsa-miR-664b-3p	0.0000	0.0000	2.45	5.45	up	5.45
hsa-miR-6728-5p	0.0094	0.0108	3.04	8.24	up	8.24
hsa-miR-6734-5p	0.0000	0.0000	4.00	16.05	up	16.05
hsa-miR-6737-3p	0.0005	0.0011	-1.39	2.62	down	-2.62
hsa-miR-6739-5p	0.0018	0.0027	-1.45	2.73	down	-2.73

hsa-miR-6743-3p	0.0000	0.0000	3.32	9.96	up	9.96
hsa-miR-6752-3p	0.0083	0.0097	-1.02	2.02	down	-2.02
hsa-miR-6753-5p	0.0000	0.0000	3.84	14.37	up	14.37
hsa-miR-6756-3p	0.0000	0.0000	2.81	6.99	up	6.99
hsa-miR-6759-3p	0.0004	0.0008	-1.52	2.87	down	-2.87
hsa-miR-6763-3p	0.0010	0.0017	-1.30	2.46	down	-2.46
hsa-miR-6766-3p	0.0019	0.0029	-1.20	2.29	down	-2.29
hsa-miR-6768-5p	0.0000	0.0000	3.29	9.79	up	9.79
hsa-miR-6772-5p	0.0000	0.0000	2.57	5.94	up	5.94
hsa-miR-6774-5p	0.0000	0.0001	-1.84	3.59	down	-3.59
hsa-miR-6777-3p	0.0033	0.0044	-1.14	2.21	down	-2.21
hsa-miR-6779-5p	0.0043	0.0056	2.86	7.27	up	7.27
hsa-miR-6785-3p	0.0005	0.0010	-1.40	2.63	down	-2.63
hsa-miR-6786-5p	0.0000	0.0000	3.08	8.43	up	8.43
hsa-miR-6788-5p	0.0000	0.0000	2.76	6.75	up	6.75
hsa-miR-6793-5p	0.0001	0.0002	2.12	4.34	up	4.34
hsa-miR-6797-3p	0.0014	0.0022	-1.38	2.60	down	-2.60
hsa-miR-6798-3p	0.0010	0.0018	-1.39	2.61	down	-2.61
hsa-miR-6800-3p	0.0008	0.0015	-1.36	2.57	down	-2.57
hsa-miR-6808-5p	0.0000	0.0000	2.58	5.97	up	5.97
hsa-miR-6809-5p	0.0070	0.0083	2.27	4.82	up	4.82
hsa-miR-6813-3p	0.0020	0.0029	-1.21	2.31	down	-2.31
hsa-miR-6819-3p	0.0039	0.0051	-1.14	2.20	down	-2.20
hsa-miR-6820-5p	0.0000	0.0000	2.55	5.86	up	5.86
hsa-miR-6824-3p	0.0009	0.0016	-1.36	2.57	down	-2.57
hsa-miR-6824-5p	0.0000	0.0000	2.75	6.71	up	6.71
hsa-miR-6829-5p	0.0000	0.0000	3.42	10.71	up	10.71
hsa-miR-6833-5p	0.0026	0.0036	2.81	7.00	up	7.00
hsa-miR-6849-5p	0.0000	0.0000	4.06	16.69	up	16.69
hsa-miR-6850-5p	0.0008	0.0015	3.34	10.14	up	10.14

hsa-miR-6851-3p	0.0009	0.0016	-1.33	2.51	down	-2.51
hsa-miR-6858-3p	0.0006	0.0011	-1.36	2.56	down	-2.56
hsa-miR-6865-3p	0.0021	0.0030	-1.25	2.38	down	-2.38
hsa-miR-6870-3p	0.0007	0.0014	-1.36	2.57	down	-2.57
hsa-miR-6879-5p	0.0012	0.0020	-1.20	2.29	down	-2.29
hsa-miR-6880-3p	0.0012	0.0020	-1.38	2.61	down	-2.61
hsa-miR-6884-3p	0.0001	0.0002	2.49	5.62	up	5.62
hsa-miR-6889-3p	0.0010	0.0017	-1.24	2.36	down	-2.36
hsa-miR-7111-3p	0.0221	0.0230	2.36	5.13	up	5.13
hsa-miR-7114-5p	0.0000	0.0000	3.25	9.49	up	9.49
hsa-miR-766-3p	0.0015	0.0023	-1.21	2.32	down	-2.32
hsa-miR-7975	0.0000	0.0001	1.97	3.91	up	3.91
hsa-miR-8060	0.0000	0.0001	2.73	6.63	up	6.63
hsa-miR-8071	0.0000	0.0000	3.18	9.09	up	9.09
hsa-miR-8089	0.0025	0.0034	2.91	7.53	up	7.53
hsa-miR-8485	0.0007	0.0013	-1.31	2.49	down	-2.49
hsa-miR-874-3p	0.0000	0.0000	2.38	5.19	up	5.19
hsa-miR-933	0.0015	0.0023	-1.25	2.38	down	-2.38
hsa-miR-98-3p	0.0271	0.0273	-1.08	2.11	down	-2.11
